# An Interacting Quantum Atoms (IQA) and Relative Energy Gradient (REG) Study of the Halogen Bond with Explicit Analysis of Electron Correlation

**DOI:** 10.3390/molecules25112674

**Published:** 2020-06-09

**Authors:** Ibon Alkorta, Arnaldo F. Silva, Paul L. A. Popelier

**Affiliations:** 1 Instituto de Química Médica (CSIC), Juan de la Cierva, 3., 28006 Madrid, Spain; ibon@iqm.csic.es; 2 Manchester Institute of Biotechnology (MIB), 131 Princess Street, Manchester M1 7DN, UK; arnsfilho@gmail.com; 3 Department of Chemistry, University of Manchester, Oxford Road, Manchester M13 9PL, UK

**Keywords:** Relative Energy Gradient (REG), Interacting Quantum Atoms (IQA), Quantum Chemical Topology (QCT), electron correlation, halogen bonding, Møller-Plesset (MP), covalency

## Abstract

Energy profiles of seven halogen-bonded complexes were analysed with the topological energy partitioning called Interacting Quantum Atoms (IQA) at MP4(SDQ)/6–31 + G(2d,2p) level of theory. Explicit interatomic electron correlation energies are included in the analysis. Four complexes combine X_2_ (X = Cl or F) with HCN or NH_3_, while the remaining three combine ClF with HCN, NH_3_ or N_2_. Each complex was systematically deformed by translating the constituent molecules along its central axis linking *X* and *N*, and reoptimising its remaining geometry. The Relative Energy Gradient (REG) method (*Theor. Chem. Acc.* **2017**, *136*, 86) then computes which IQA energies most correlate with the total energy during the process of complex formation and further compression beyond the respective equilibrium geometries. It turns out that the covalent energy (i.e., exchange) of the halogen bond, X…N, itself drives the complex formation. When the complexes are compressed from their equilibrium to shorter X…N distance then the intra-atomic energy of *N* is in charge. When the REG analysis is restricted to electron correlation then the interatomic correlation energy between X and N again drives the complex formation, and the complex compression is best described by the destabilisation of the through-space correlation energy between N and the “outer” halogen.

## 1. Introduction

The halogen bond [1,2,3,4] is the second oldest non-covalent interaction after the hydrogen bond. According to the IUPAC definition [5] a halogen bond occurs when there is evidence of a net attractive interaction between an electrophilic region (sometimes referred to as the σ-hole [6]) associated with a halogen atom in a molecular entity and a nucleophilic region in another, or the same, molecular entity. It is known [7] that σ-holes are electron-deficient regions arising from the anisotropic distribution of electron density on the atoms of Group 14, 15, 16 and 17 elements when covalently bonded to electron-withdrawing groups yielding non-covalent bonds named as tetrels, pnictogens, chalcogens and halogens, respectively.

The nature of the halogen bond interaction has been disputed for a long time. It is interesting that, already in the 1950s, Mulliken explained the color of a solution of I_2_ in different electron-donating solvents based on an intermolecular charge transfer process during light absorption [8]. The more recent literature describes how several energy partitioning methods have been applied to halogen bonded complexes. The conclusion reached depends on the energy partitioning method and the set of systems considered. In particular, Shaik and coworkers applied valence bond theory and the block-localised wave functions to 55 neutral complexes and polyhalogen anions, and concluded that in most species the dominant term is charge transfer [9,10]. A similar conclusion was drawn by Infante et al. using natural energy decomposition analysis (NEDA), and by Head-Gordon et al. using energy decomposition analysis (EDA), for complexes of CX_3_I (X = F, Cl, Br and I) [11,12]. Using both the natural bond orbital method and the Quantum Theory of Atoms in Molecules, Grabowski found [13] that halogen bonds and hydrogen bonds are ruled by the same mechanisms involving charge transfer, hyperconjugation and rehybridisation effects, while the electrostatic interaction drive the initiation and directionality. 

Other work disputes the importance of charge transfer. Řezáč and de la Lande, using constrained DFT, quantified charge transfer as 10% on average of the interaction energy [14]. Joubert et al. explored neutral and anionic complexes with the Quantum Theory of Atoms in Molecules (QTAIM) [15] and the Interacting Quantum Atom (IQA) [16] method at HF and DFT level [17]. They found that the primary interaction is electrostatic but the exchange contribution between the main atoms is the dominant term in the equilibrium geometry [18]. Riley and coworkers have applied Symmetry-Adapted Perturbation Theory (SAPT) to different sets of complexes and showed that electrostatics and dispersion are the most important attractive terms [19,20,21]. Using the SAPT (DFT) method, Stone showed that the binding is usually electrostatically driven but the equilibrium geometries are not always determined by electrostatics alone. In particular, the strong tendency to linearity of the B···XY bond is a consequence of exchange–repulsion, not electrostatics [22]. However, it should be noted that the SAPT method does not include an explicit charge transfer term. Cremer et al. studied 36 halogen-bonded complexes YX⋯AR*_m_* (X = F, Cl, Br; Y = donor group; AR*_m_* = acceptor group) at CCSD (T) level [23]. These authors concluded that both electrostatic and covalent contributions are important. They showed that the covalent character of the halogen bond increases as the three-center four-electron bond became possible.

The extensive efforts described above were mainly focused on the dichotomy between electrostatics and quantum effects via several theoretical paradigms. However, it is known [20,24] that dispersion plays an important role in the formation of the halogen bond. A good example of this role is the short distance, low-polarity, contacts between Cl and Cl in Cl_3_C–CCl_3_⋯Cl_3_C–CCl_3_. These interactions differ from dihalogen bonds because there is no alignment of the σ-hole with a region of negative charge on the other halogen [25]. Recently, more attention has been given to the evaluation of the role of dispersion over the stability of systems, mostly due to advances in theory and implementation of non-bonded interactions. Our group, in particular, has been applying some of its software development and hardware resource into developing a new way of including dispersive forces in chemical systems by means of the IQA decomposition [26,27,28,29,30,31,32,33]. Our methods enable the evaluation of the correlation energy in real-space, i.e., we can determine the correlation energy between any two atoms in the system and within any atomic basin. The IQA partition is also available within the context of coupled cluster theory, [34] coupled-cluster Lagrangian densities [35] and CCSD(T) wave functions [36]. The treatment of pure electron correlation, outside of a DFT framework, is computationally very expensive and hence limits the number of systems we can analyse in this article.

In this article, seven halogen-bonded complexes with binding energies between 6 and 40 kJ mol^−1^ have been investigated. The dissociation profile of the complexes has been calculated at MP4 (SDQ) level. These results have been analysed using IQA and the Relative Energy Gradient (REG) [37] method including a term that takes into account the correlation contribution, which contains the coveted dispersion contribution. REG is minimalistic unbiased procedure to compute chemical insight by ranking individual IQA energy contributions by importance. In particular, REG contrasts the latter with the total energy of the system as the system undergoes a chemical process of interest (e.g., hydrogen bond formation [37], rotation barrier [38] or S_N_2 reaction [39]). We aim to contribute with an analysis, for the first time within the context of REG/IQA, of the role of correlation/dispersion energy in the energetic make-up of halogen-bonded complexes. Our work is different from a previous REG/IQA study [40] on halogen bonding in at least 3 ways: (i) different complexes, (ii) DFT (B3LYP, hence no pure electron correlation) *versus* post-Hartree-Fock ab initio method (MP4(SDQ)), and (iii) angular analysis (directionality) *versus* translational analysis (formation and compression of complex). Fairly recently Tognetti and Joubert carried out extensive computational work^2^ at B3LYP-D2 level of theory using QTAIM and IQA by systematically varying the X…N distance in Cl_3_COX…NH_3_ complexes (X = F, Cl, and Br). Our work differs from theirs, not only in the complexes looked at, but also in the way dispersion was handled: in their own words, the “D2 procedure actually provides a correction to the DFT approximations in order to recover the correct interaction energies for reference van der Waals systems, and not a direct evaluation of the dispersion energy.” In the current work we evaluate the dispersion directly.

## 2. Results and Discussion

### 2.1. Minima Configurations

Seven halogen-bonded complexes for which the experimental geometry was determined using rotational spectroscopy have been selected for the current study. The small number of complexes investigated is determined by the prohibitive computational cost of the MP4(SDQ)-IQA method. These complexes combine three halogen bond donors: F_2_, Cl_2_ and ClF, with three nitrogen bases: HCN, NH_3_ and N_2_, but obviously not exhaustively. The calculated intermolecular distances nicely resemble the experimental ones, with maximum deviations between −0.02 and +0.07 Å, where a negative value means that a calculated value underestimates the experimental one. The calculated binding energies of the complexes range between −39.6 kJ mol^−1^ (for H_3_N:ClF, the second strongest in a large number of complexes studied [41]) and −5.5 kJ mol^−1^ (for HCN:F_2_). Table 1 summarises these results.

**Figure 1:**
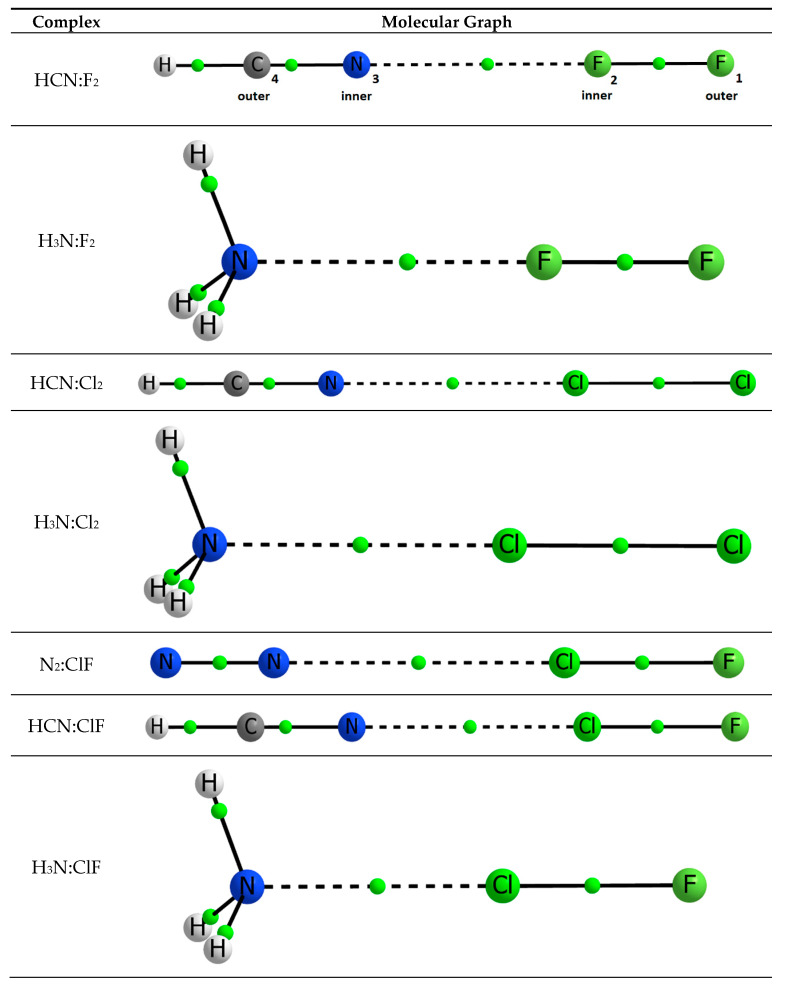
Molecular graphs of all seven complexes studied. The complex HCN:F_2_ has been marked to illustrate the labelling throughout the paper. The location of each BCP is marked by a small green sphere.

The topological analysis of the electron density shows the presence of a single intermolecular Bond Critical Point (BCP) between the two interacting moieties along the symmetry axis. Figure 1 shows the molecular graphs of the least stable complex (top) and the most stable one (bottom). 

Table 2 collects the values of three densities evaluated at the intermolecular BCP (ρ_BCP_, ∇^2^ρ_BCP_ and H_BCP_), which are typical of weak non-covalent interactions for all the complexes. The only exception is the most stable complex (H_3_N:ClF) where the negative value of H_BCP_ indicates [49] a partial covalent character of the interaction. Recently Cremer et al. [23] studied 36 halogen-bonded complexes at CCSD(T)/aug-cc-pVTZ level of theory of which three were studied in this article, namely H_3_N:F_2_, H_3_N:Cl_2_ and H_3_N:ClF. Their respective values for ρ_BCP_ were 0.014, 0.025 and 0.053 a.u., which are close to the corresponding values in Table 2. Similarly, their respective values for H_BCP_ were 0.004, 0.001 and −0.009 a.u., which agree with the values in Table 2 but in sign and order of magnitude only. Stronger complexes (with P in the accepting moiety) can have H_BCP_ values that are up to an order of magnitude larger than seen here. 

### 2.2. Energy Profiles

Using the equilibrium geometry as reference, we changed, in regular steps and up to a distance of 4 Å, the intermolecular distance in each complex between the two atoms involved in the interaction (and connected to the intermolecular BCP). In addition, distances shorter than the equilibrium were considered too. The properties of the intermolecular BCP along the dissociation profile were analysed independently for the two N···F contacts and the five N···Cl contacts. In both sets, ρ_BCP_ and ∇^2^ρ_BCP_ increase as the intermolecular distance decreases and follow an exponential relationship. The Laplacian values, obtained for N···Cl contacts close to 2.1 Å, show the location of the maximum of this property, and confirms previous reports [50]. The total energy density, H_BCP_, steadily increases as the N···F distance decreases, while in the N···Cl contacts, H_BCP_ increases up to around 2.7 Å but for shorter distances tends to become smaller and even negative for values smaller than 2.4 Å (see Figure S2).

Level 1: Total Atomic Energies 

Table S2 shows the complete REG analysis of the energy profile at the first level considered (i.e., total atomic energies) for all seven complexes. Table 3 lists the most positive and most negative REG value in each complex for both segments. In principle, a REG analysis of only its positively-valued energy contributions may suffice to explain what drives the formation of a complex. However, in order to expand the REG analysis, a complementary explanation can be drawn from the most *negative* REG values. Such an alternative explanation can sometimes be more intuitive. A second and very important criterion for deriving a good explanation from a REG analysis is the uniformity across all complexes. Indeed, the most powerful explanation is one that encompasses all complexes. Finally, we point out that an explanation is always linked to the direction of analysis: from left to right, or *vice versa*. However, we note that the relationship between the profile of E_i_ and that of E_tot_ is actually intrinsic, that is, independent of the direction of the explanation. Sometimes it is more natural to explain a segment from right to left, sometimes from left to right. Throughout this work we will always analyse a segment from right to left. For example, the segment between the equilibrium distance and infinity (SEG2) is best thought of as the formation of a complex (rather than its destruction), because one usually wonders which type of force pulls the monomers together. Similarly, it also makes sense to see SEG1 as a compression energy profile, rather than one relaxing from a very short-range complex to the equilibrium one.

**Figure 2:**
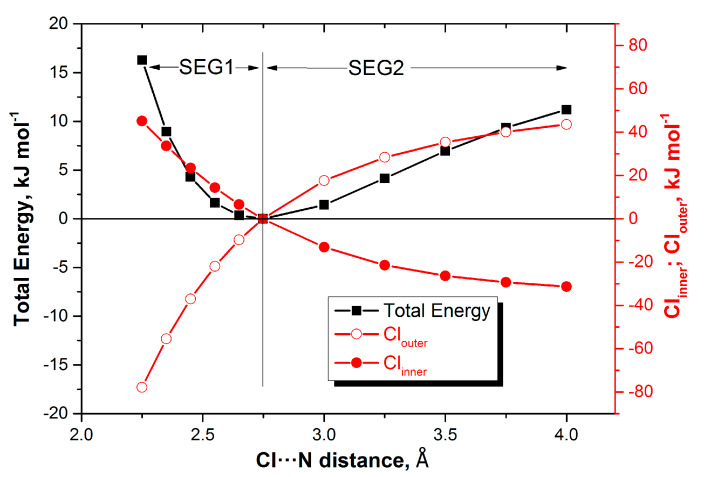
Energy profiles (total system, total atomic Cl_outer_ and total atomic Cl_inner_) of H_3_N:Cl_2_ as a function of the distance between the “inner contact atoms” Cl_inner_ and N_3_. The three energies all refer to the corresponding energies at equilibrium, referring to the respective energy scales (left = total system, right = atomic energies).

We first look for overall patterns emerging from Table 3. One such pattern is that the atoms, one for each complex, associated with the most positive REG values of SEG1, are the same as those associated with the most negative REG values of SEG2. This finding means that the two segments mirror each other in the following sense. For each complex, the atom that most works *against* the total energy profile (negative REG) in one segment, also works most *with* the total energy (positive REG) in the other segment. Figure 2 makes this behaviour clear for one representative complex (H_3_N:Cl_2_). Here, atom Cl_inner_ works most *with* the total energy in SEG1, and very much *against* the total energy in SEG2, so in each complex, there is a single atom that fulfills such an overall role over the whole energy profile. The same is true for Cl_outer_ but then vice versa. 

Still, one could question the chemical and practical significance of finding such a single atom explaining the *whole* energy profile, without segmentation. Indeed, there is another, more fruitful way of looking at Figure 2, but then focusing only on the positive REG values. Figure 2 shows that the coming together of H_3_N and Cl_2_, from infinity to equilibrium (SEG2), is governed by the total atomic stabilisation of the outer Cl. Indeed, this atom has the most positive REG values in SEG2 (see Table 3). On the other hand, in SEG1, H_3_N and Cl_2_ are brought together further and compressed beyond the equilibrium geometry. This time, the total energy profile is ruled by the total atomic destabilisation of the other Cl, i.e., the inner Cl, directly involved in the halogen bond. So, which energy contribution, either in SEG1 or SEG2, that best explains the total energy profile is learnt from the most positive REG value, respectively. 

We look again at Table 3 and focus on the positive REG values only. When the X_2_ complexes are formed (SEG2) then X_outer_ dominates. On the other hand, when complexes with F_2_ and Cl_2_ are compressed (SEG1) then the total atomic energy of X_inner_ dominates. These conclusions can also be drawn Figure 2, which shows a X_2_ complex. However, the three complexes with ClF reveal a more complex picture than for the four X_2_ complexes. It is difficult to summarise the observations. However, the clearest common thread is the role of the “contact N” or inner nitrogen (i.e., N_3_), whose stabilisation drives the formation of all three complexes (SEG2). The compression of the complexes (SEG1), up the repulsive barrier of the total energy profile, is predominately caused by non-contact atoms in the partner molecule of ClF. This finding can be conveniently depicted by the bold underlined atoms in **N**N:ClF, H**C**N:ClF and **H_3_**N:ClF. 

In summary, we have learnt that: (i) the formation of X_2_ complexes is driven by the stabilisation of the outer X, but (ii) their compression by the inner X, while (iii) the formation of ClF complexes is driven by the stabilisation of the contact N, and (iv) their compression by non-contact atoms in the partner molecule of ClF. 

Level 2: Total Interatomic and Intra-Atomic Energies

The next levels of REG analysis operate on more resolved energy data and may help to shed light on the nature of the (de)stablisation of the total atomic energies. Table S2 shows the REG analysis at Level 2 (total interatomic and intra-atomic energies). From Table S2, Table 4 now extracts the *two* most important terms instead of only one, as in Table 3. This relaxation serves the purpose of ideally recovering a single explanation across all complexes. In other words, allowing a second close competitor to take the role of the most important contribution increases the chance of generating a uniform interpretation across complexes. Since there are now many more energy contributions to consider than at Level 1, REG values should be forced to be higher than a certain threshold, which was set to 2.0. Few complexes did not meet this threshold causing the absence of a second entry in Table 4.

We analyse the whole energy profile from right to left, starting with SEG2. The X_inner_–N_inner_ interaction is the major driving force (i.e., positive REG) to complex formation. In other words, the two contact atoms at the heart of each complex exert the “clearest pull”. This observation is true for all seven complexes, so there is no need to distinguish the X_2_ complexes from the ClF complexes, as had to be done at Level 1. Moving further to the left we then study the compression segment (SEG1). This time the intra-atomic energy of the inner nitrogen (N_inner_ or N_3_) governs the total energy barrier. Chemically, this finding means that it is the steric “discomfort” of the contact N that explains the compression energy barrier. Note that in order to reach this conclusion for all seven complexes we had to invoke the second most important term for one complex. 

**Figure 3:**
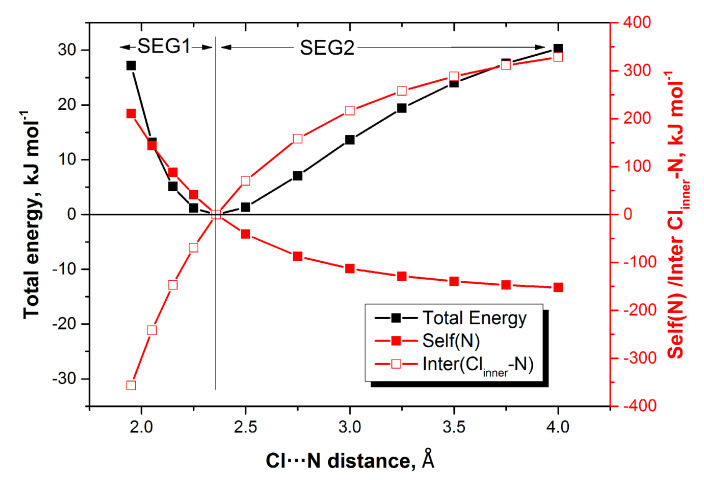
Energy profiles (total, intra-atomic energy of N_3_, and interatomic Cl_inner_–N_3_) of H_3_N:ClF as a function of the distance between the “inner contact atoms” Cl_inner_ and N_3_.

Interpreting the positive REG values suffices to understand the chemical behaviour of the complexes but for completeness one may inspect the most negative REG values as well. The good news is again that one single type of energy contribution is in charge for all complexes without exception: the interatomic X_inner_–N_inner_ energy. We have to wait for the more resolved analysis of Level 3 to know which type of energy is behind X_inner_–N_inner_ but, whichever its nature, it vehemently works against the compression energy barrier. Figure 3 shows the evolution of the intra-atomic energy of N_inner_ and that of the X_inner_–N_inner_ energy alongside the total energy profile of NH_3_:ClF.

In summary, at Level 2 we have learnt that, for all seven complexes, (i) the interatomic X_inner_–N_inner_ energy drives their formation (i.e., energy of contact atom…contact atom), and that (ii) their compression is dominated by the destabilisation of N_inner_ (i.e., contact N).

Before we move on the next level of REG analysis, Level 3, we briefly contrast Level 2 and Level 1. Level 2 firmly associates the driving forces for both formation and compression with the inner (contact) atoms, and *does so for all seven complexes*. In contrast, the total atomic energy analysis of Level 1, brings in non-contact atoms as drivers and differentiates two different types of complex (X_2_ and ClF). So, the explanation at Level 2 is more minimal than that at Level 1. In short, Level 2 is thus more powerful, and therefore preferred. We deduce that a too coarse REG analysis (Level 1) spoils the uniformity of interpretation and thus its power.

Level 3: Total Intra-Atomic (E_intra_), Electrostatic (V_cl_), Exchange (V_x_) and Correlation (V_c_) Energies.

It remains to be seen if the even more resolved Level 3 analysis can repeat the uniformity of Level 2 but now also reveal the type of interaction energy. Table S4 gathers all data in connection with Level 3, which is the most resolved (i.e., the least coarse) REG analysis that we apply here. Level 3 takes into account four possible types of energy: $E_{\mathrm{intra}}^{A}$, the classical electrostatic energy $V_{\mathrm{cl}}^{AB}$, the exchange energy $V_{x}^{AB}$, and the correlation energy $V_{c}^{AB}$. Similarly to Level 2, Table 5 skims from Table S4 the *two* most important energy terms, by absolute REG value.

Again we analyse the whole energy profile from right to left, starting with SEG2, and focus on the most positive values. We find that the covalent energy contribution V_x_ (X_inner_–N_inner_) is dominant. This is true for all seven complexes although for HCN:ClF the third most positive REG value had to be invoked, as the two energy terms with higher REG values were electrostatic in nature. Note that there is a sudden drop (Table S4) in REG value after the third term, which we allowed to slip in order to make the interpretation uniform throughout all complexes. How can we contextualise the conclusion that the predominant interaction forming a halogen-bonded complex is covalent in nature?

It is instructive to briefly look at hydrogen bonding first. Our first ever REG study illustrated this newly proposed method^37^ on the water dimer, in which two monomers form the global energy minimum. In this classic hydrogen-bonded system, REG shows that the formation of the dimer is dominated by the electrostatic energy between the hydrogen-bond hydrogen atom and the acceptor oxygen. All subsequent terms are also electrostatic in nature, thereby confirming the well-known Buckingham-Fowler model [51]. However, the sixth term in that sequence is of the type exchange-correlation (as the study was carried out using DFT). The exchange energy typically dominates the correlation energy in this combined energy term, which means that there is some covalent character in a hydrogen bond.

Now, Wolters and Bickelhaupt compared [52] hydrogen-bonded systems with halogen-bonded ones from a molecular orbital perspective. From their extensive analysis on DX…A^−^ and on DH…A^−^ (D, X, A = F, Cl, Br, I) systems they concluded that halogen bonds and hydrogen bonds have a very similar bonding mechanism consisting of both electrostatic and covalent contributions. However, they also stated that the electrostatic attraction is less favourable in the halogen bonds, adding that halogen bonds can become stronger than hydrogen bonds because of a more stabilising covalent component in the former. In summary, their conclusions agree with our own. To strengthen this main point, we mention that Li et al. concluded [53] from their combined QTAIM/NBO study on complexes with formaldehyde and hypohalous acids that the energy decomposition analyses indicate that the contribution from the electrostatic interaction energy is larger in the hydrogen-bonded complexes than that in the halogen-bonded complexes. Next, in the rather vague and future-work-directed conclusions of their review of computer modelling of halogen bonds, Kolář and Hobza unfortunately do not offer the insight that we need to support our own findings or not. Still, on the basis of S66 and X40 benchmark data sets, they mention a difference between hydrogen-bonded and halogen-bonded systems as decided by the SAPT scheme. Whereas the electrostatic energy is the most attractive contribution to hydrogen-bonded systems, they state, the situation is different for halogen-bonded systems. For the latter, they quote a medley of energy types being responsible, short of declaring exchange though. In short, the clarity that REG offers is not matched with any statements found in this review.

The next discussion point is how the dominance of covalent energy is compatible with the σ-hole model, which is of course electrostatic in nature. Let us take the example of H_3_N:ClF to understand how REG operates. Figure S3 directly contrasts V_x_ (X_inner_–N_inner_) and V_cl_ (X_inner_–N_inner_), first for the full complex formation energy (SEG2, eight distances) and then for the long-range part of that segment (four longest distances). For the full segment it is clear that the electrostatic linear fit is better (R^2^ = 0.99) than that of the exchange fit (R^2^ = 0.88). However, the REG value (i.e., slope) of the exchange interaction is higher (5.33) than that of the electrostatic interaction (4.47). So, in spite of the worse fit, the exchange is singled out as the most pronounced interaction aiding the total energy profile. However, the situation flips for the long-range where the REG value (4.44) of the electrostatic interaction now dominates that of the exchange (2.08), with both fits being excellent (R^2^ ~ 0.99). This REG-based conclusion is compatible with the σ-hole interpretation of electrostatics being in charge at long-range.

However, it is clear that a complex closer to its equilibrium geometry is *not* governed by electrostatics. In fact, a fit of the 4 shortest distances returns a REG value of 9.41 (R^2^ = 0.91) for exchange and 5.34 (R^2^ = 0.95) for electrostatics. Indeed, at short-range the X_inner_–N_inner_ interaction becomes overwhelmingly covalent in nature, as the exchange REG value is then almost twice that over the whole SEG2 interval, and almost five times that at long-range. Tognetti and Joubert mention^2^ the “dual character” of the halogen bond, classical electrostatics being comparable to covalency. For their Cl_3_COX…NH_3_ complexes (X = F, Cl, and Br) they used IQA (but not REG) and QTAIM to show that electrostatics generally predominates at long-range, and that exchange becomes an important contribution around the equilibrium position.

Finally, we contrast the REG findings, which are based on global properties (i.e., obtained by integration over atomic volumes) with local properties (i.e., evaluated at a point, e.g., ρ_BCP_, ∇^2^ρ_BCP_ and H_BCP_ listed in Table 2). The local properties suggest that halogen bonds are non-covalent but the REG analysis suggests that their behaviour at short-range (stretching the complex beyond the equilibrium by a modest amount) is covalent. There is a case to make (also done by others) that global properties are more trustworthy than local ones because they carry more information (given the volume integration, which covers a multitude of points). Otherwise, one may question to what extent the BCP properties describe the same entity as the REG-IQA data. The former is a quick characterisation of the nature of a bond in the minimum energy geometry only. This is a static picture that contrasts with the dynamic nature of REG, on top of the global nature of IQA. The REG-IQA data may point at an energy term that poorly describes, drives or explains a whole energy segment of complex formation, such as the electrostatic energy. But then, this term can have a significant value at the equilibrium geometry, and be linked to the non-covalency (i.e., “closed-shell interaction”) indicted by the local properties.

After this lengthy discussion of the nature of the SEG2 energy profile, we now look at the compression regime (SEG1). We attempt to find an explanation, common to all complexes, typically starting with the positive REG values. If we allow flipping between the most and second most important term, then again “Intra-n3” or the total intra-atomic energy of N_inner_ is the dominant term. This finding is compatible with the conclusion of Level 2.

**Figure 4:**
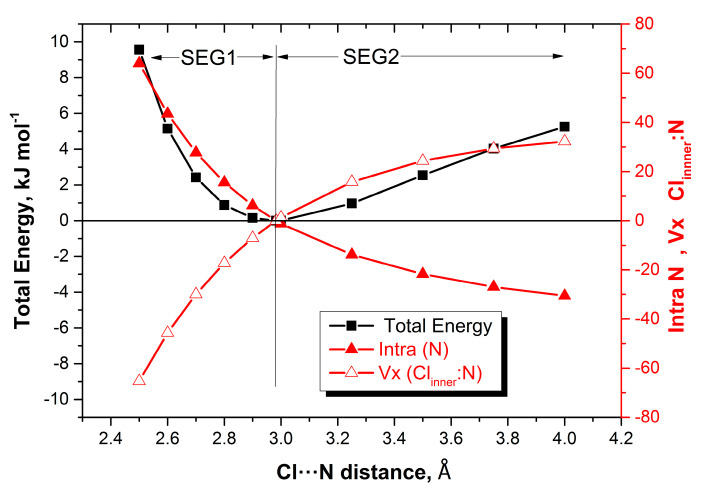
Energy profiles (total, intra-atomic energy of Cl_inner_, and interatomic exchange V_x_(Cl_inner_–N_3_)) of HCN:Cl_2_ as a function of the distance between the “inner contact atoms” Cl_inner_ and N_3_.

Equally good news emerges when looking at the most negative REG values. They show a clear pattern for all seven complexes and highlight V_x_ (X_inner_–N_inner_) as the dominant energy. Thus, the covalent energy between the halogen atom and the Lewis base contact atom most opposes the compression. Level 2 already identified this interaction but could not determine its type. However, now we can, at Level 3. Indeed, Figure 4 shows how this interaction emphatically lowers its energy upon compression of HCN:Cl_2_ and therefore strongly opposes the compression energy barrier.

In summary, at Level 3 and for all seven complexes, we find that: (i) the exchange energy between X_inner_ and N_inner_ drives their formation, and that (ii) their compression is best described by the destabilisation of N_inner_ (i.e., contact N).

Level 4: Correlation/Dispersion Energies Only

We now move on to the final level of analysis. None of the correlation terms (V_c_) plays a significant role in explaining the total energy profile because of their small absolute REG values in Table S4. Still, given the attention that electron correlation received in the literature we can confine a REG analysis to correlation terms only. This is done in the fourth and final level of REG analysis, Level 4, which focuses only on electron correlation and thus dispersion. It is true that the correlation energy contribution seems to be small or relatively meaningless, compared to the much larger REG values seen for V_cl_ or V_x_. Still, one should remember that the binding energies of the halogen complexes studied here (and in general) are relatively small, ranging from 5.5 to 39.6 kJ mol^−1^, compared to those of covalent bonds. This means that if one fails to take into account the electron correlation contribution, the halogen bond could potentially cease to exist altogether, especially for those compounds at the weaker end of the energy spectrum. We now turn our sights to the correlation/dispersion part of the halogen bond invoking a final REG analysis.

Table 6 gives the REG analysis of the correlation (V_c_) terms only, both intra- and interatomic. As always, we discuss the complex formation regime (SEG2) first. The most important positive REG contribution is V_c_ (X_inner_–N_inner_), i.e., the interaction between the two (contact) atoms involved in the halogen bond. This result is “clean” in the sense that there was no need to involve the second most important REG contribution, and V_c_ (X_inner_–N_inner_) showed up for all seven complexes. So, the correlation contribution mirrors that of the exchange, V_x_ (X_inner_–N_inner_), as shown in Level 3. Of course the exchange energy overwhelms the correlation one but if the latter is excluded (as it is in Table 6) then correlation would be responsible for forming the complex. 

For sake of completeness Table 6 also lists the most negative REG values where, curiously, V_c_ (X_outer_–N_inner_) now dominates for all complexes (with the exception of N_2_:ClF where the second most important contribution had to be invoked). This is a “through space” effect that is hard to interpret but it returns based on the most positive REG values for the compression segment (SEG1). In other words, if we focus on electron correlation only (and thus omit the intra-atomic interaction of N_inner_) then the destabilisation of the correlation energy between X_outer_ and N_inner_ best explains the repulsive compression energy barrier. For completeness we add that V_c_ (X_inner_–N_inner_) most opposes the compression (with the only exception of H_3_N:ClF). In other words, this contribution keeps becoming more stable as the complex is compressed.

A further remark concerns the importance of dispersion in halogen bonding. A study [54] used DFT-SAPT on benzene…dihalogen and formaldehyde…compound halogen-bonded complexes, covering X…π and Cl…O contacts amongst others. It turned out that the dispersion energy is non-negligible and indeed of the same order of magnitude as the electrostatic energy. This statement should not be confused with our own findings and wrongly deemed to be contradictory. Earlier work^27^ from our lab showed that the interatomic correlation energies of halogen bonds and hydrogen bonds are on a par, and of the order of 1 to 3 kJ mol^−1^. This contribution is not negligible (see Table 2) and similar to that found between the hydrogens in a single water molecule. Moreover, the intra-atomic correlation energy within a hydrogen in a single water is about 20 kJ mol^−1^. The intra-atomic components are typically much larger than the interatomic ones, which is also true for the helium dimer. As a consequence, we showed^26^ that the stability of this traditional van der Waals complex is best explained by (intra)atomic correlation energy lowering, rather than by a direct dispersion interaction between the helia. So, we are not claiming that the various electron correlation contributions in halogen-bonded systems are negligible. However, we do claim that the interatomic correlation energies cannot be used to explain what drives the formation or repulsive compression energy barrier of a complex, when these energies operate in the presence of much larger contributions such as exchange or steric energy (intra-atomic energy).

In summary, we learn from the exclusive electron correlation REG analysis at Level 4 that (i) the interatomic correlation energy between X_inner_ and N_inner_ drives the complex formation, and that (ii) complex compression is best described by the destabilisation of the through-space correlation energy between X_outer_ and N_inner_.

Finally, we comment briefly on charge transfer, which is well-defined within the topological approach, which does not introduce any reference states and is parameter-free. Figure S4 profiles for all seven complexes. The overall net charge of the halogen molecule is plotted versus the N…X distance, for the whole distance range (SEG1 and SEG2). It is convenient to express the charge transfer in milli-electron (m*e*), a resolution well supported by the atomic integration error, which is typically two to three orders of magnitude smaller. It is clear that all halogen molecules (ClF, F_2_, Cl_2_) start off almost neutral (except in N_2_:ClF where a very small positive charge built up, of less than 1 m*e*). Subsequently, a negative net charge accumulates as each complex forms, increasing in magnitude to respectively −8, −22, −5, −9, −3, −18 and −11 m*e* for HCN:F_2_, H_3_N:F_2_, HCN:Cl_2_, H_3_N:Cl_2_, N_2_:ClF, HCN:ClF and H_3_N:ClF. There is no correlation between the binding energy of the complex and its molecular charge transfer. Note that this charge transfer peaks (in absolute value) at the shortest complex distance, at values that can be about two to six times larger than at equilibrium.

The reason we mention charge transfer data here is because of the attention it received from researchers using non-topological energy decomposition analyses. For example, Thirman et al. argued [11] that a picture of the halogen bond that excludes charge transfer cannot be complete. Based on their study on systems of the type CX_3_I…Y^−^ (for X = F, Cl, Br, I and Y = F, Cl, Br) they went on to state that permanent and induced electrostatics do not always provide the dominant stabilising contributions to halogen bonds. Their work was triggered by an earlier study [12] on these systems that showed that the trend in binding strength is exactly opposite to the trend determined by σ-hole size. From the point of view of the current study, charge transfer is connected to the electrostatic energy as the first term of its multipolar expansion. In that sense, charge transfer is only part of a more complete narrative based on the electrostatic energy itself, which already played a full role in our REG-IQA analysis. Indeed, we could link the excess charge on the halogen molecule (and the corresponding depletion on the other monomer in the complex) to a crude estimate of the electrostatic energy between the monomers. However, this severe approximation and the fact that there is no visible trend between the numerical values of charge transfer and the binding energy (see above), makes this an avenue not worth pursuing. Instead, we used the REG-IQA analysis itself to point out that the σ-hole model can be linked to the underlying atomic energy patterns only at long-range (N…Cl distances ≥3.25 Å in the H_3_N:ClF complex). The importance of the covalent character of the halogen bond, that we discovered in the current study, automatically weakens the trustworthiness of the σ-hole when used at short-range. 

## 3. Computational Methods

### 3.1. Energy Profiles

The geometry of the systems has been optimised at MP4(SDQ) level [55] with the fully uncontracted 6–31 + G(2d,2p) basis set [56]. We imposed C_3v_ symmetry on the complexes containing NH_3_, and C_∝v_ for the remaining complexes, in agreement with the described experimental geometries of the systems studied. Starting from the respective equilibrium intermolecular distance of each system, this distance was increased in steps of 0.25 Å for values larger than the equilibrium up to a maximum interatomic distance of 4 Å, while optimizing the rest of the geometry. Similarly, intermolecular distances smaller than the equilibrium distance, were decreased in steps of 0.10 Å while maintaining the initial symmetry. The total number of configurations ranged between 10 and 13 depending on the system considered. The shape of the energetic profiles for all systems is shown in Figure S1 of the Supporting Information. All calculations were carried out with the GAUSSIAN16 program [57]. 

### 3.2. Interacting Quantum Atoms (IQA)

This method has been reviewed many times (e.g., refs. [38,58]) so we confine this subsection to enough revision to understand the symbols marking the IQA energy contributions used in the Results and Discussion section, and define the levels at which the REG analyses are organised. We start from the coarsest partitioning used in this work, which is one at atomic level. At this level, called Level 1, we associate a single energy for each atom *A*, $E_{\mathrm{IQA}}^{A}$, without revealing the (full quantum mechanical) nature of this energy (electrostatic, exchange, correlation, kinetic…). We can then obviously write:

(1) $$E_{total}=\sum_{A}E_{\mathrm{IQA}}^{A}$$

where the energy of the system, *E_total_,* should ideally be exactly equal to the sum of the atomic energy. However, in reality, there is an error due to the challenge of atomic integration, which is quantified in the next subsection.

The next, finer level of partitioning (Level 2), splits the total atomic energy into intra-atomic terms, $E_{\mathrm{intra}}^{A}$ and interatomic terms, $V_{\mathrm{inter}}^{AB}$, as shown in Equation (2), which also provides the link between Levels 1 and 2:

(2) $$E_{\mathrm{IQA}}^{A}=E_{\mathrm{intra}}^{A}+V_{\mathrm{inter}}^{A}=E_{\mathrm{intra}}^{A}+\frac{1}{2}\sum_{B\neq A}V_{\mathrm{inter}}^{AB}$$

Level 2 operates with two types of energy, $E_{\mathrm{intra}}^{A}$ and $V_{\mathrm{inter}}^{AB}$, which do not reveal the full quantum mechanical nature of the energy. However, the next level of analysis, Level 3, does. 

Level 3 is the most detailed (or resolved) level that we will consider but not the most detailed possible. The latter would involve intra-atomic exchange energy and kinetic energy, which are harder to interpret in terms of typical chemical language, if at all. Level 3 thus consists of four energy types, in which $E_{\mathrm{intra}}^{A}$ is kept while $V_{\mathrm{inter}}^{AB}$ is split into three parts: the classical electrostatic energy $V_{\mathrm{cl}}^{AB}$, the exchange energy $V_{x}^{AB}$, and the correlation energy $V_{c}^{AB}$. Note that the correlation energy is typically the smallest in absolute value. The term $V_{\mathrm{cl}}^{AB}$ is introduced because the Coulomb term is typically not analysed as a pure electron-electron energy term ($V_{\mathrm{ee},\mathrm{coul}}^{AB}$) but is combined with potential energies involving the nuclei of atoms *A* and *B*, or:

(3) $$V_{\mathrm{cl}}^{AB}=V_{ee,coul}^{AB}+V_{en}^{AB}+V_{ne}^{AB}+V_{nn}^{AB}$$

where $V_{ne}^{AB}$ is the energy associated with the nucleus of atom *A* interacting with the electron density of atom *B*, and $V_{nn}^{AB}$ is the nucleus-nucleus repulsion.

Two comments on Level 3 are worth making. One concerns the link between Level 3 and Level 2, which is given by Equation (4):

(4) $$V_{\mathrm{inter}}^{AB}=V_{\mathrm{cl}}^{AB}+V_{x}^{AB}+V_{c}^{AB}$$

The other comment concerns the chemical nature of $E_{\mathrm{intra}}^{A}$, which gather all intra-atomic terms:

(5) $$E_{\mathrm{intra}}^{A}=T^{A}+V_{cl}^{AA}+V_{x}^{AA}+V_{c}^{AA}$$

where T^A^ is the kinetic energy and, $V_{\mathrm{cl}}^{AA}$, the intra-atomic counterpart of $V_{\mathrm{cl}}^{AB}$ is simply given by:

(6) $$V_{\mathrm{cl}}^{AA}=V_{ee,coul}^{AA}+V_{en}^{AA}$$

There is a practical reason that the four energy intra-atomic energy types are condensed into $E_{intra}^{A}$. We have shown before [59,60] that there is a quantitative link between steric repulsion and $E_{\mathrm{intra}}^{A}$ via the latter’s successful fitting to the well-known exponential Buckingham potential. This potential is superior to the Lennard-Jones one, which fortunately did not fit so well to $E_{intra}^{A}$. Moreover, combination rules were recovered allowing repulsive interactions between atoms *A* and *B* to be derived from such interactions between *A* and *A* on one hand, and *B* and *B* on the other. So, although the most resolved level would involve seven energy types (*T^A^*, $V_{cl}^{AA}$, $V_{x}^{AA}$, $V_{c}^{AA}$, $V_{cl}^{AB}$, $V_{x}^{AB}$ and $V_{c}^{AB}$) this analysis is not applied in this work because it does not lead to a chemically satisfactory picture. Finally, Level 4 focuses on the electron correlation/dispersion only covering both intra- and interatomic contributions.

### 3.3. Partitioning of Electron Correlation

The wavefunction has been divided in two parts. The first part corresponds to the Hartree-Fock contribution and the second one to the correlation contribution. Thus, the IQA analysis of the wavefunction has been carried out in two steps. In the first step, the Hartree-Fock part of the wavefunction was processed with the program AIMAll [61]. In the second step, the correlation part was treated by the program MORFI, which is an in-house derivative of MORPHY [62]. The atomic integration error, calculated as the difference between the sum of the IQA terms of the Hartree-Fock wavefunction and the original ab initio energy, is always very small (less than 0.1 kJ mol^−1^) as shown in Table S1. In contrast, the errors of the correlation contributions are much larger (up to 4.6 kJ mol^−1^ in absolute value). However, the profiles of the calculated MP4 (SDQ) energies and the sum of all the IQA terms (HF and correlation) are very similar for all systems.

We have explained the MPn-IQA method in detail before [26] and will therefore not repeat it here. Equation (1) shows how this approach divides up the two-particle density-matrix into single-atom (intra-atomic) terms and atom-atom (interatomic) terms, where the latter can refer to any two atoms *A* and *B*, not necessarily bonded ones:

(7) $$V_{c}^{AB}=\sum_{j=1}^{N_{basis}}\sum_{k=1}^{j}K_{jk}\sum_{l=1}^{N_{basis}}\sum_{m=1}^{l}K_{lm}d_{jklm}^{corr}\int_{\Omega_{A}}dr_{1}G_{jk}(r_{1}-R_{jk})\int_{\Omega_{B}}dr_{2}\frac{1}{r_{12}}G_{lm}(r_{2}-R_{lm})$$

where V_c_ is the (electron) correlation energy. Note that *A* can be equal to *B*, which then corresponds to the intra-atomic correlation energy. Numerical integration is carried out over the volumes of two topological atoms (Ω_A_ and Ω_B_), with *d^corr^* representing the two-particle density-matrix. The subscripts *j*, *k*, *l* and *m* define the origins of the *N_basis_* Gaussian (primitive) basis functions. For each two Gaussian basis functions indexed *j* and *k*, there is a constant *K_jk_* that follows from applying the Gaussian product theorem to their pre-factors.

### 3.4. The Relative Energy Gradient (REG) Method

The REG method has also been explained many times elsewhere [37,38,39,40,63,64,65] and will thus not be repeated in the same detail here. REG is associated with a dynamic change to the system under study. The method thus needs a sequence of “snapshots” of the system in order to display to what extent a given IQA energy term’s profile follows the total energy profile. This sequence is governed by the control coordinate *s*, which is in our case the intermolecular distance between the moieties making up the van der Waals complexes. REG then looks for correlations, occurring along this sequence, between the total energies and the atomic energies. The energy profile is separated into *segments* that are defined by the turning points in the total energy. In this work there are two segments: one between the complex’s equilibrium distance and the shortest distance considered (SEG1), and the other segment between the equilibrium distance and the largest distance considered (SEG2). REG analyses each segment separately, and relates the gradient (denoted *m_REG_*, dimensionless) of a partitioned energy (denoted *E_i_*) to the total energy of the system (denoted *E_total_*) by linear regression:

(8) $$E_{i}(s)=m_{REG_{i}}\cdot E_{total}(s)+c_{i}$$

where $m_{REG_{i}}$ (or the “REG value”) is calculated by ordinary least squares and c_i_ is the intercept. Note that (i) there is an equation like Equation (8) for each energy term *i*, and that it is fitted to the energy data points that represent the segment, and that (ii) the energies *E_total_* and *E_i_* are actually shifted by subtracting from them their respective mean energies. The absolute value of the Pearson correlation coefficient (associated with the regression) should be as close as possible to unity and many values are indeed above 0.95. Once the REG values have been calculated they are ordered from most positive to most negative (i.e., largest to smallest). A positive REG value means that the partitioned energy gradient acts in the same direction as the total energy over the given segment. In other words, the partitioned energy contribution behaves similarly to the total energy. The opposite is true for a negative REG value. The REG method ranks all IQA energy terms such that the terms with the largest magnitude REG values are more chemically relevant than IQA terms with smaller magnitude REG values. Due to its exhaustive nature, the REG method detects all effects, no matter how subtle, and ranks them quantitatively. The REG analysis was carried out with the ANANKE program [37], which acts on the calculated points of the energy profile, and enables an analysis at the four levels described above. In all cases, the MP4(SDQ)/6–31 + G(2d,2p) energy has been used as *E_total_* (Equation (8)). 

## 4. Conclusions

IQA is a modern energy partitioning method that delivers intra-atomic and interatomic energy contributions that are well-defined, particularly at short-range. When combined with MP4(SDQ), IQA generates electron correlation contributions via in-house software for halogen-bonded complexes with a binding energy between −6 and −40 kJ mol^−1^. Due to the computational expense of the six-dimensional atomic integrations operating alongside the typically enormous two-particle density-matrix, only 7 complexes could be analysed. The equilibrium geometries of these complexes were systematically compressed leading to a repulsive energy barrier; they were also elongated to study the formation of the complex from its constituent monomers. Even for the small systems studied here, IQA already generates dozens of energy contributions. To cope with the numerous energies and their change during the two energy profiles studied the REG method steps in. This minimal method singles out which type of energy contribution (steric, electrostatic, exchange, correlation) and which atoms involved, best describe the total energy profile. The most resolved level of analysis shows that (i) the exchange energy between X_inner_ and N_inner_ drives the complex formation, and that (ii) the complexes’ compression is best described by the destabilisation of N_inner_, which is essentially steric energy. If the REG analysis is confined to correlation energy only then it turns out that (i) the interatomic correlation energy between X_inner_ and N_inner_ drives the complex formation, and that (ii) complex compression is best described by the destabilisation of the through-space correlation energy between X_outer_ and N_inner_.

## Figures and Tables

**Table 1:** Experimental and calculated intermolecular distances (*Å*) and calculated binding energies (kJ mol^−1^).

Complex	N⋯X Dist. Exp.	N⋯X Dist. Calc.	Binding Energy
HCN:F_2_	2.803 [42]	2.794	−5.5
H_3_N:F_2_	2.708 [43]	2.684	−8.5
HCN:Cl_2_	2.915 [44]	2.982	−9.6
H_3_N:Cl_2_	2.724 [45]	2.748	−17.7
N_2_:ClF	2.920 [46]	2.969	−6.9
HCN:ClF	2.639 [47]	2.684	−18.8
H_3_N:ClF	2.376 [48]	2.359	−39.6

**Table 2:** Intermolecular BCP properties (all in a.u. except the distance).

	N··X Distance (Å)	ρ_BCP_	∇^2^ρ_BCP_	H_BCP_
HCN:F_2_	2.794	0.008	0.039	0.002
H_3_N:F_2_	2.684	0.013	0.053	0.002
HCN:Cl_2_	2.983	0.010	0.044	0.002
H_3_N:Cl_2_	2.748	0.021	0.070	0.002
N_2_:ClF	2.969	0.009	0.042	0.002
HCN:ClF	2.684	0.019	0.079	0.002
H_3_N:ClF	2.359	0.047	0.137	−0.003

**Table 3:** REG analysis of the total atomic energies $E_{\mathrm{IQA}}^{A}$ (Level 1). For each complex, two atoms are listed, that with the most positive and that with the most negative REG value (dimensionless, see Equation (8)). The REG values are shown in parentheses. The atomic labeling follows X_outer_–X_inner_ ⋯ N_3_- etc. where X = Cl or F, and the numeric labels of atoms not marked in this core common scheme increase while moving away from X_inner_.

**SEG1**						
**HCN:F_2_**	**H_3_N:F_2_**	**HCN:Cl_2_**	**H_3_N:Cl_2_**	**N_2_:ClF**	**HCN:ClF**	**H_3_N:ClF**
F_inner_	F_inner_	Cl_inner_	Cl_inner_	N4	C4	H *
(2.94)	(3.54)	(2.95)	(2.56)	(1.55)	(2.33)	(1.35)
F_outer_	F_outer_	Cl_outer_	Cl_outer_	F_outer_	F_outer_	Cl_inner_
(−3.08)	(−3.72)	(−3.40)	(−4.49)	(−1.64)	(−1.27)	(−1.70)
**SEG2**						
**HCN:F_2_**	**H_3_N:F_2_**	**HCN:Cl_2_**	**H_3_N:Cl_2_**	**N_2_:ClF**	**HCN:ClF**	**H_3_N:ClF**
F_outer_	F_outer_	Cl_outer_	Cl_outer_	N3	N3	N3
(3.60)	(4.00)	(3.55)	(3.49)	(1.50)	(2.29)	(1.38)
F_inner_	F_inner_	Cl_inner_	Cl_inner_	N4	C4	H *
(−3.34)	(−3.74)	(−2.78)	(−2.51)	(−2.42)	(−2.38)	(−0.84)

* Contribution of any of the three symmetry-equivalent H atoms.

**Table 4:** REG analysis of the total interatomic, $V_{\mathrm{inter}}^{AB}$, and intra-atomic energies, $E_{\mathrm{intra}}^{A}$ (Level 2). For each complex, the largest two energies are listed (most positive and most negative REG value by absolute value except if smaller than 2.0). The REG values are shown in parentheses. The atomic labeling follows X_1_–X_2_ ⋯ N_3_- etc. where X = Cl or F, and the numeric labels of atoms not marked in this core common scheme increase while moving away from X_inner_.

**SEG1**						
**HCN:F_2_**	**H_3_N:F_2_**	**HCN:Cl_2_**	**H_3_N:Cl_2_**	**N_2_:ClF**	**HCN:ClF**	**H_3_N:ClF**
Intra_n3	Intra_n3	Intra_cl2	Intra_n3	Intra_n3	Intra_n3	Intra_n3
(6.36)	(6.65)	(6.50)	(7.78)	(5.56)	(7.81)	(7.15)
Intra_f2	Intra_f2	Intra_n3	Intra_cl2	Intra_cl2	Inter_cl2_c4	Inter_f1_cl2
(6.27)	(6.14)	(6.35)	(6.30)	(4.59)	(4.01)	(4.09)
Intra_f1	Intra_f1	Inter_n3_c4	Intra_cl1		Inter_n3_c4	Intra_f1
(−3.39)	(−5.04)	(−2.58)	(−4.97)		(−3.25)	(−2.17)
Inter_f2_n3	Inter_f2_n3	Inter_cl2_n3	Inter_cl2_n3	Inter_cl2_n3	Inter_cl2_n3	Inter_cl2_n3
(−9.22)	(−10.34)	(−11.35)	(−13.74)	(−8.71)	(−12.71)	(−12.11)
**SEG2**						
**HCN:F_2_**	**H_3_N:F_2_**	**HCN:Cl_2_**	**H_3_N:Cl_2_**	**N_2_:ClF**	**HCN:ClF**	**H_3_N:ClF**
Inter_f2_n3	Inter_f2_n3	Inter_cl2_n3	Inter_cl2_n3	Inter_cl2_n3	Inter_cl2_n3	Inter_cl2_n3
(6.47)	(7.46)	(11.87)	(10.54)	(9.93)	(13.03)	(9.80)
Intra_f1	Intra_f1	Inter_n3_c4	Intra_cl1		Inter_n3_c4	
(3.48)	(4.17)	(4.34)	(3.58)		(4.35)	
Intra_n3	Intra_n3	Intra_n3	Intra_n3	Intra_n3	Intra_n3	Inter_f1_n3
(−2.62)	(−3.25)	(−5.72)	(−4.91)	(−2.82)	(−4.89)	(−2.04)
Intra_f2	Intra_f2	Intra_cl2	Intra_cl2	Intra_cl2	Inter_cl2_c4	Intra_n3
(−5.93)	(−6.55)	(−6.60)	(−5.95)	(−4.86)	(−5.81)	(−4.41)

**Table 5:** REG analysis (level 3) of the total intra-atomic energy, $E_{\mathrm{intra}}^{A}$, and inter-atomic energies, by all types: electrostatic (V_cl_), exchange (V_x_) and correlation (V_c_). For each complex, the largest two energies are listed (most positive and most negative REG value by absolute value except if smaller than 2.0). The REG values are shown in parentheses. The atomic labeling follows X_1_–X_2_ ⋯ N_3_- etc. where X = Cl or F, and the numeric labels of atoms not marked in this core common scheme increase while moving away from X_2_ (X_inner_).

**SEG1**						
**HCN:F_2_**	**H_3_N:F_2_**	**HCN:Cl_2_**	**H_3_N:Cl_2_**	**N_2_:ClF**	**HCN:ClF**	**H_3_N:ClF**
Intra_n3	Vx_f1_f2	Intra_cl2	Intra_n3	Intra_n3	Intra_n3	Intra_n3
(6.36)	(6.80)	(6.50)	(7.78)	(5.56)	(7.81)	(7.15)
Intra_f2	Intra_n3	Intra_n3	Intra_cl2	Intra_cl2	Vcl_cl2_c4	Vx_f1_cl2
(6.27)	(6.65)	(6.35)	(6.30)	(4.59)	(4.22)	(4.43)
Intra_f1	Intra_f1	Vcl_cl2_n3	Vcl_cl2_n3	Vcl_f1_cl2	Vcl_cl2_n3	Vcl_cl2_n3
(−3.39)	(−5.04)	(−4.91)	(−5.52)	(−2.44)	(−5.07)	(−3.61)
Vx_f2_n3	Vx_f2_n3	Vx_cl2_n3	Vx_cl2_n3	Vx_cl2_n3	Vx_cl2_n3	Vx_cl2_n3
(−7.20)	(−8.06)	(−6.43)	(−8.22)	(−7.61)	(−7.64)	(−8.50)
**SEG2**						
**HCN:F_2_**	**H_3_N:F_2_**	**HCN:Cl_2_**	**H_3_N:Cl_2_**	**N_2_:ClF**	**HCN:ClF**	**H_3_N:ClF**
Vx_f2_n3	Vx_f2_n3	Vx_cl2_n3	Vx_cl2_n3	Vx_cl2_n3	Vcl_cl2_n3	Vx_cl2_n3
(4.81)	(5.65)	(6.07)	(5.80)	(6.98)	(7.93)	(5.33)
Intra_f1	Intra_f1	Vcl_cl2_n3	Vcl_cl2_n3	Vcl_cl2_n3	Vcl_n3_c4 ^a^	Vcl_cl2_n3
(3.48)	(4.17)	(5.80)	(4.74)	(2.92)	(5.50)	(4.47)
Intra_n3	Intra_n3	Intra_n3	Intra_n3	Intra_n3	Intra_n3	Vx_f1_cl2
(−2.62)	(−3.25)	(−5.72)	(−4.91)	(−2.82)	(−4.89)	(−2.49)
Intra_f2	Intra_f2	Intra_cl2	Intra_cl2	Intra_cl2	Vcl_cl2_c4	Intra_n3
(−5.93)	(−6.55)	(−6.60)	(−5.95)	(−4.86)	(−5.93)	(−4.41)

^a^ The 3rd most positive REG value is Vx_cl2_n3, with value of 5.10 (while the 4th one has the much smaller value of 3.38, see Table S4). Note that Vx_cl2_n3 is in line with the expected V_x_ (X_inner_–N_inner_).

**Table 6:** REG analysis of the correlation (V_c_) terms only, both intra- and interatomic. For each complex, the largest two energies are listed (most positive and most negative REG value by absolute value). The REG values are shown in parentheses. The atomic labeling follows X_1_–X_2_ ···N_3_^−^ etc. where X = Cl or F, and the numeric labels of atoms not marked in this core common scheme increase while moving away from X_inner_.

**SEG1**						
**HCN:F_2_**	**H_3_N:F_2_**	**HCN:Cl_2_**	**H_3_N:Cl_2_**	**N_2_:ClF**	**HCN:ClF**	**H_3_N:ClF**
Vc_f1_n3	Vc_f1_n3	Vc_cl1_n3	Vc_cl1_n3	Vc_f1_n3	Vc_f1_n3	Vc_cl2_n3
(0.18)	(0.21)	(0.11)	(0.13)	(0.12)	(0.09)	(0.07)
						Vc_f1_n3
						(0.07)
	Vc_f2_n3		Vc_cl2_n3			Vc_cl2_h4
	(−0.43)		(−0.25)			(−0.04)
Vc_f2_n3	Vc_f1_f2	Vc_cl2_n3	Vc_cl1_cl2	Vc_cl2_n3	Vc_cl2_n3	Vc_f1_cl2
(−0.75)	(−0.43)	(−0.41)	(−0.33)	(−0.69)	(−0.40)	(−0.33)
**SEG2**						
**HCN:F_2_**	**H_3_N:F_2_**	**HCN:Cl_2_**	**H_3_N:Cl_2_**	**N_2_:ClF**	**HCN:ClF**	**H_3_N:ClF**
Vc_f2_n3	Vc_f2_n3	Vc_cl2_n3	Vc_cl2_n3	Vc_cl2_n3	Vc_cl2_n3	Vc_cl2_n3
(0.89)	(0.60)	(0.88)	(0.45)	(1.05)	(0.57)	(0.24)
				Vc_f1_n3		
				(−0.22)		
Vc_f1_n3	Vc_f1_n3	Vc_cl1_n3	Vc_cl1_n3	Vc_n3_n4	Vc_f1_n3	Vc_f1_n3
(−0.27)	(−0.18)	(−0.17)	(−0.10)	(−0.29)	(−0.10)	(−0.07)

## Supplementary Materials

The following are available online, Figure S1: Energy profiles calculated at MP4(SDQ)/6–31 + G(2d,2p) level for all 7 complexes, Figure S2: Evolution of ρBCP,∇2ρBCP and HBCP (in a.u.) along the energy profiles of all 7 complexes, Figure S3: Relation between the total energy of H3N:ClF (relative to the energy minimum, in Hartree), and the electrostatic (red) and exchange (blue) energy contributions (relative to the energy minimum, in Hartree) for the (top) full complex formation energy segment (SEG2) and the (bottom) long-range-only part of the complex formation segment, Figure S4: Charge transfer profiles for all 7 complexes. The overall net charge of the halogen molecule in plotted (in e) versus the N…X distance (in Å). Table S1: Energy range of the energy profiles and the corresponding (atomic integration) errors calculated as the difference between the sum of the IQA terms and the original ab initio energy (kJ mol^−1^), Table S2: REG analysis of the energy profiles of all seven complexes based on the total atomic energies ranked by their REG values, Table S3: REG analysis of the energy profile of all seven complexes based on the total interatomic and intra-atomic energies ranked by their REG values, Table S4: REG analysis of the energy profile of all seven complexes based on all the atomic energies electrostatic (Vcl), exchange (Vx) and correlation (Vc), ranked by their REG values.
