# Supplementary material for: An Interacting Quantum Atoms (IQA) and Relative Energy Gradient (REG) Study of the Halogen Bond with Explicit Analysis of Electron Correlation
Source: Molecules. 2020 Jun 9;25(11):2674. doi: 10.3390/molecules25112674 (PMC7321288; doi:10.3390/molecules25112674)

Supporting Information

Interacting Quantum Atoms (IQA) and Relative Energy Gradient (REG) Study of the Halogen Bond with explicit Analysis of Electron Correlation

Ibon Alkorta, Arnaldo F. Silva and Paul L. A. Popelier

Index:

Pg. 2-4 **Figure S1.** Energy profiles calculated at MP4(SDQ)/6-31+G(2d,2p) level for all 7 complexes.

Pg. 5 **Table S1.** Energy range of the energy profiles and the corresponding (atomic integration) errors calculated as the difference between the sum of the IQA terms and the original *ab initio* energy (kJ mol^{-1}).

Pg. 6 **Figure S2.** Evolution of ρ_{BCP},$\nabla^2\rho_{BCP}$ and H_{BCP} (in a.u.) along the energy profiles of all 7 complexes.

Pg. 7-8 **Table S2**. REG analysis of the energy profiles of all seven complexes based on the **total atomic energies** ranked by their REG values.

Pg. 9-12 **Table S3.** REG analysis of the energy profile of all seven complexes based on the **total interatomic and intra-atomic energies** ranked by their REG values

Pg. 13-20 **Table S4.** REG analysis of the energy profile of all seven complexes based **on all the atomic energies electrostatic (V_{cl}), exchange (V_x) and correlation (V_c)**, ranked by their REG values.

Pg. 21 **Figure S3.** Relation between the total energy of H_3N:ClF (relative to the energy minimum, in Hartree), and the electrostatic (red) and exchange (blue) energy contributions (relative to the energy minimum, in Hartree) for the (top) **full** complex formation energy segment (SEG2) and the (bottom) **long-range-only** part of the complex formation segment.

Pg. 22 **Figure S4.** Charge transfer profiles for all 7 complexes. The overall net charge of the halogen molecule in plotted (in e) versus the N…X distance (in Å).

Figure S1. Energy profiles calculated at MP4(SDQ)/6-31+G(2d,2p) level for all 7 complexes.

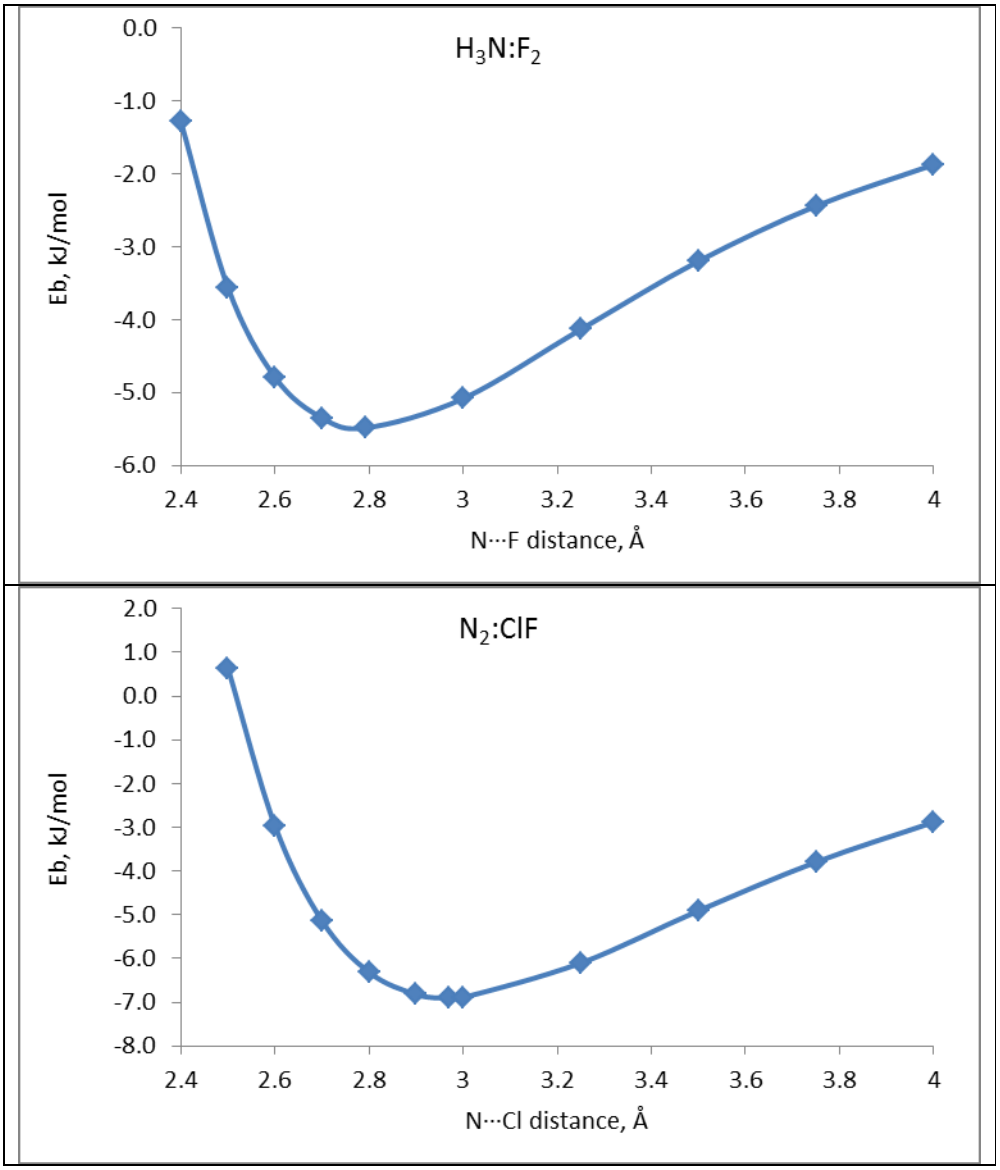

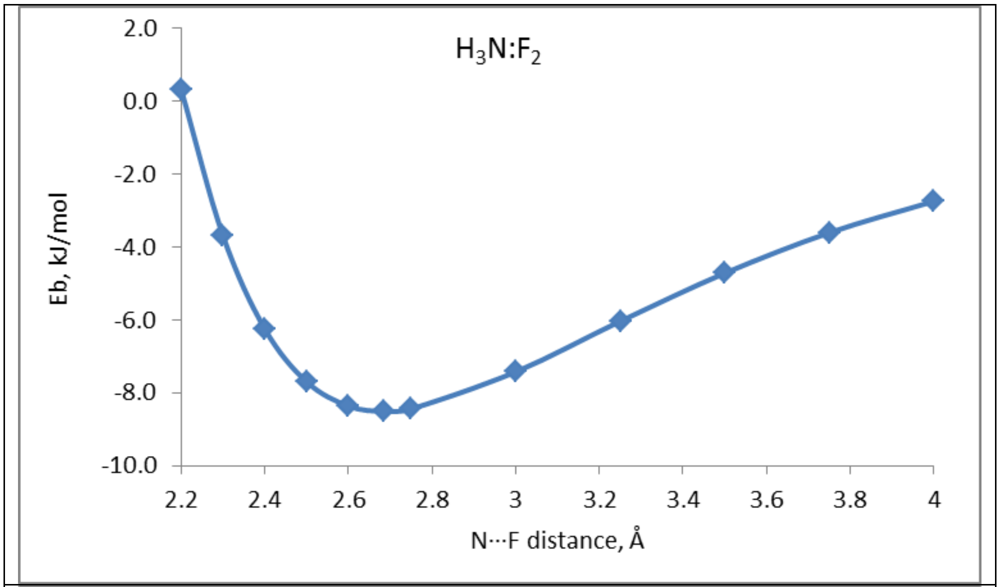

$H_3N:F_2$
Eb, kJ/mol
2.0
0.0
-2.0
-4.0
-6.0
-8.0
-10.0
2.2
2.4
2.6
2.8
3
3.2
3.4
3.6
3.8
4
N···F distance, Å

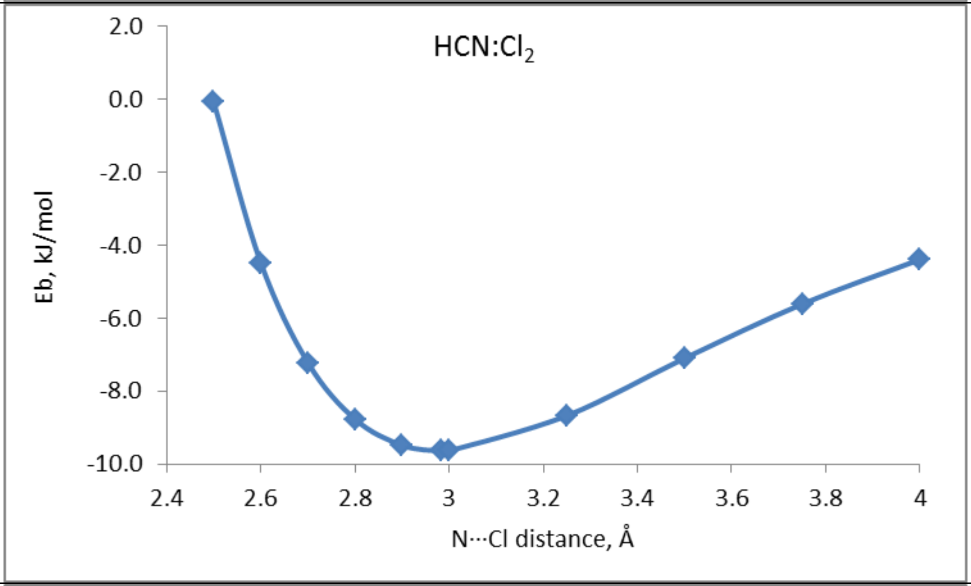

$HCN:Cl_2$
Eb, kJ/mol
2.0
0.0
-2.0
-4.0
-6.0
-8.0
-10.0
2.4
2.6
2.8
3
3.2
3.4
3.6
3.8
4
N···Cl distance, Å

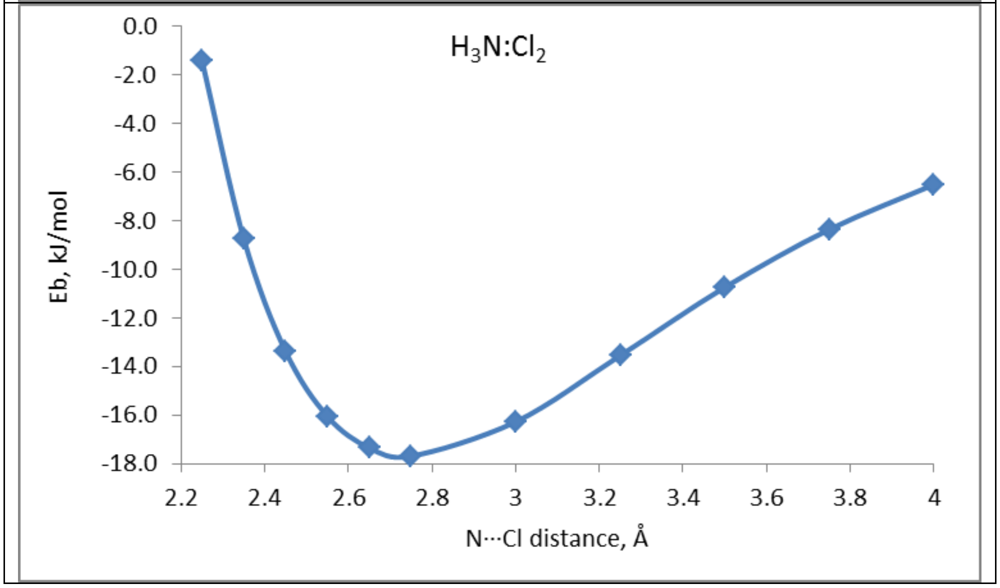

$H_3N:Cl_2$
Eb, kJ/mol
0.0
-2.0
-4.0
-6.0
-8.0
-10.0
-12.0
-14.0
-16.0
-18.0
2.2
2.4
2.6
2.8
3
3.2
3.4
3.6
3.8
4
N···Cl distance, Å

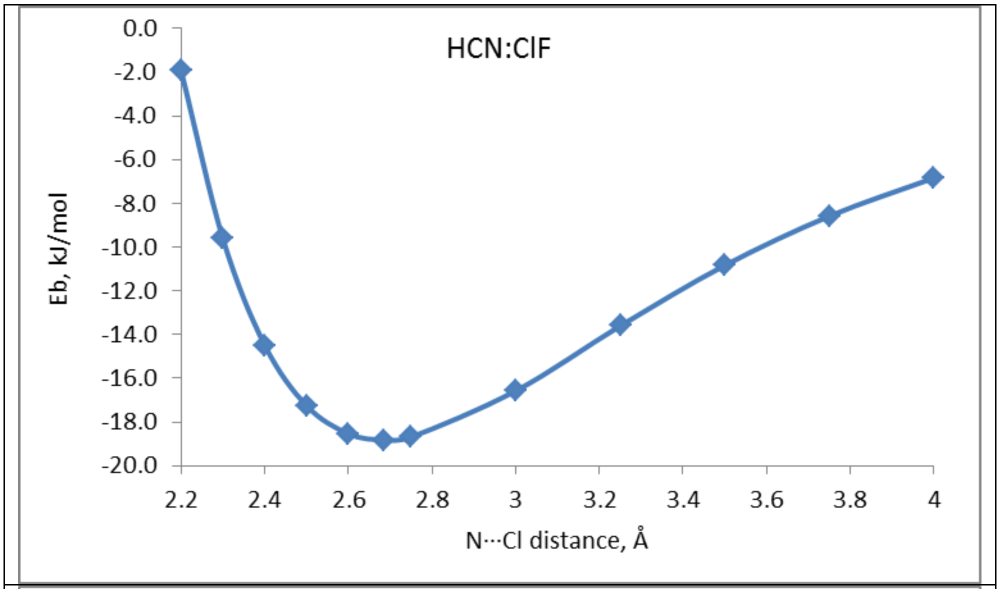
HCN:ClF
Eb, kJ/mol
0.0
-2.0
-4.0
-6.0
-8.0
-10.0
-12.0
-14.0
-16.0
-18.0
-20.0
2.2
2.4
2.6
2.8
3
3.2
3.4
3.6
3.8
4
N···Cl distance, Å

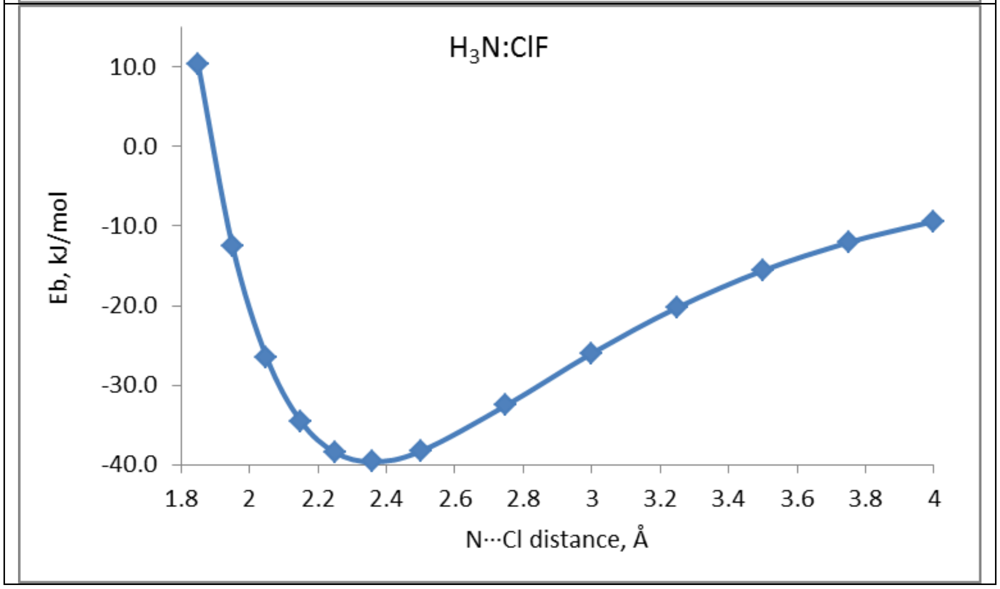
H3N:ClF
Eb, kJ/mol
10.0
0.0
-10.0
-20.0
-30.0
-40.0
1.8
2
2.2
2.4
2.6
2.8
3
3.2
3.4
3.6
3.8
4
N···Cl distance, Å

Table S1. Energy range of the energy profiles and the corresponding (atomic integration) errors calculated as the difference between the sum of the IQA terms and the original *ab initio* energy (kJ mol^{-1}).

**Complexes**	**Energy Range**	**Hartree-Fock energy**		**Correlation contribution**	
		**Max-Error**	**Min-Error**	**Max-Error**	**Min-Error**
$HCN:F_2$	4.2	0.07	0.06	2.0	1.6
$H_3N:F_2$	8.8	0.07	0.05	-0.2	-0.9
$HCN:Cl_2$	9.6	0.00	-0.02	-3.0	-4.6
$H_3N:Cl_2$	9.6	0.00	-0.03	3.3	2.2
$N_2:ClF$	7.6	0.02	0.00	-0.7	-3.0
HCN:ClF	16.9	0.04	0.02	-1.9	-4.7
$H_3N:ClF$	30.2	0.08	-0.03	-1.0	-4.5

Figure S2. Evolution of ρ_{BCP}, $\nabla^2\rho_{BCP}$ and H_{BCP} along the energy profiles of all seven complexes.

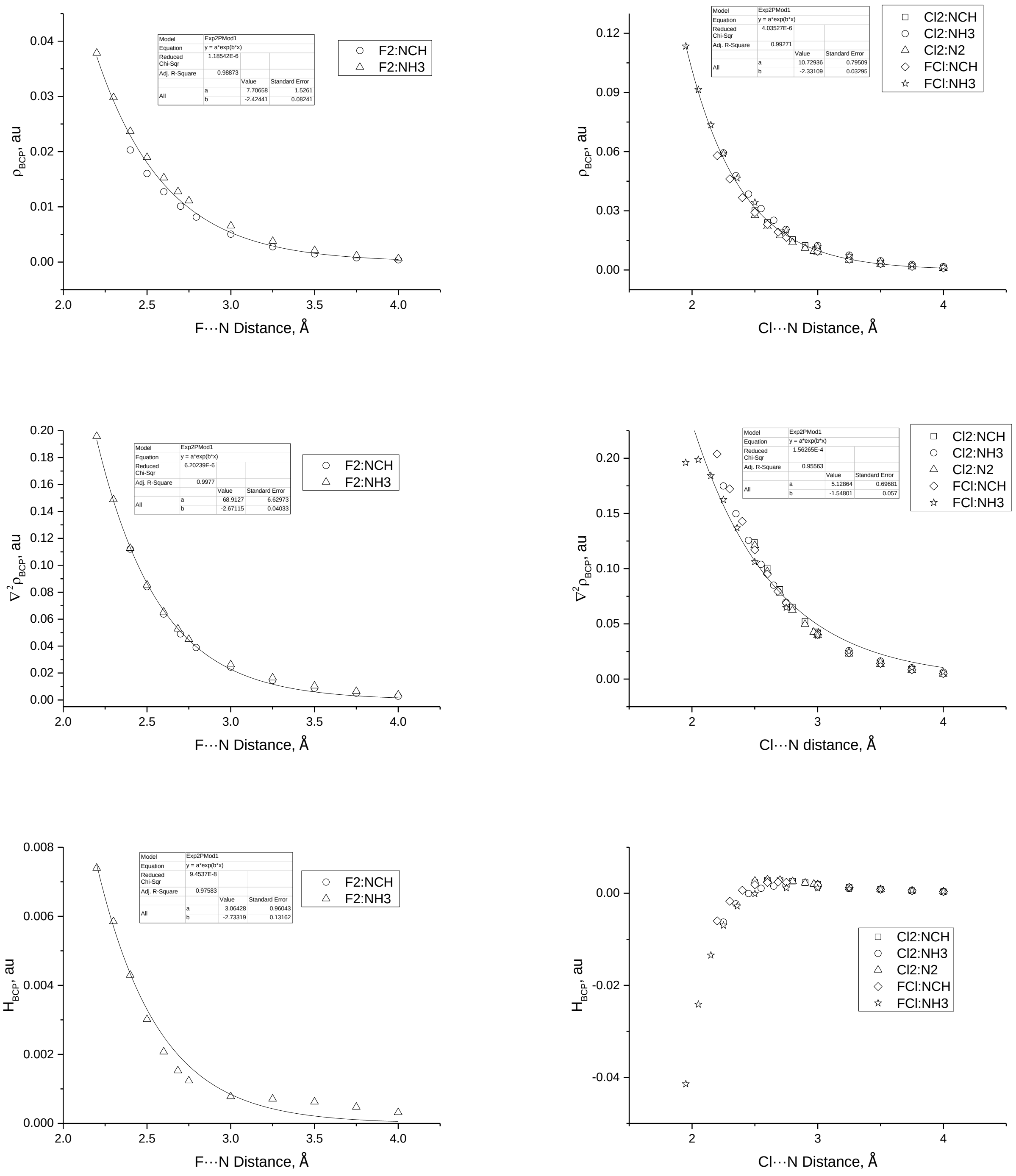

Table S2

Level 1 REG analysis of the energy profiles of all seven complexes based on the **total atomic energies** (E_{IQA}^{A}) ranked by their REG values. The atomic labelling should be clear from the common core scheme X1-X2···N3-etc. Note that in Table 3 of the main text, which summarises this Table, X1 is referred to as X_{outer}, and X2 as X_{inner}.

HCN:F_2

SEG1				SEG2		
**TERM**	**REG**	**R**		**TERM**	**REG**	**R**
EIQA_f2	2.94	0.97		EIQA_f1	3.60	0.98
EIQA_n3	1.87	0.99		EIQA_n3	1.02	0.98
EIQA_c4	0.78	0.96		EIQA_h5	-0.23	-0.99
EIQA_h5	0.17	0.97		EIQA_c4	-1.25	-0.99
EIQA_f1	-3.08	-0.96		EIQA_f2	-3.34	-0.98

H_3N:F_2

SEG1				SEG2		
**TERM**	**REG**	**R**		**TERM**	**REG**	**R**
EIQA_f2	3.54	0.97		EIQA_f1	4.00	0.97
EIQA_n3	1.83	0.99		EIQA_n3	0.28	0.70
EIQA_h5	0.72	1.00		EIQA_h5	-0.36	-0.97
EIQA_h4	0.72	1.00		EIQA_h6	-0.36	-0.97
EIQA_h6	0.72	1.00		EIQA_h4	-0.36	-0.97
EIQA_f1	-3.72	-0.98		EIQA_f2	-3.74	-0.97

HCN:Cl_2

SEG1				SEG2		
**TERM**	**REG**	**R**		**TERM**	**REG**	**R**
EIQA_cl2	2.95	0.97		EIQA_cl1	3.55	0.97
EIQA_c4	1.92	0.97		EIQA_n3	2.50	0.99
EIQA_h5	0.45	0.97		EIQA_h5	-0.56	-0.98
EIQA_n3	-0.07	-0.22		EIQA_c4	-2.50	-0.97
EIQA_cl1	-3.40	-0.98		EIQA_cl2	-2.78	-0.95

$H_3N:Cl_2$

SEG1				SEG2		
**TERM**	**REG**	**R**		**TERM**	**REG**	**R**
EIQA_cl2	2.56	0.96		EIQA_cl1	3.49	0.95
EIQA_h6	1.35	0.98		EIQA_n3	1.70	0.98
EIQA_h4	1.35	0.98		EIQA_h6	-0.79	-0.94
EIQA_h5	1.35	0.98		EIQA_h5	-0.79	-0.94
EIQA_n3	-0.17	-0.72		EIQA_h4	-0.79	-0.94
EIQA_cl1	-4.49	-0.97		EIQA_cl2	-2.51	-0.94

$N_2:ClF$

SEG1				SEG2		
**TERM**	**REG**	**R**		**TERM**	**REG**	**R**
EIQA_n4	1.55	0.97		EIQA_n3	1.50	1.00
EIQA_n3	1.41	1.00		EIQA_f1	1.24	0.94
EIQA_cl2	0.55	0.96		EIQA_cl2	-0.27	-0.71
EIQA_f1	-1.64	-0.98		EIQA_n4	-2.42	-0.98

HCN:ClF

SEG1				SEG2		
**TERM**	**REG**	**R**		**TERM**	**REG**	**R**
EIQA_c4	2.33	0.98		EIQA_n3	2.29	1.00
EIQA_h5	0.60	0.98		EIQA_f1	0.88	0.94
EIQA_n3	0.48	0.97		EIQA_cl2	0.23	0.98
EIQA_cl2	-0.30	-0.99		EIQA_h5	-0.57	-0.98
EIQA_f1	-1.27	-0.97		EIQA_c4	-2.38	-0.98

$H_3N:ClF$

SEG1				SEG2		
**TERM**	**REG**	**R**		**TERM**	**REG**	**R**
EIQA_h6	1.35	0.97		EIQA_n3	1.38	1.00
EIQA_h5	1.35	0.97		EIQA_cl2	0.89	0.92
EIQA_h4	1.35	0.97		EIQA_f1	0.68	0.95
EIQA_n3	-0.03	-0.68		EIQA_h6	-0.84	-0.95
EIQA_f1	-0.86	-0.96		EIQA_h5	-0.84	-0.95
EIQA_cl2	-1.70	-0.95		EIQA_h4	-0.84	-0.95

Table S3

Level 2 REG analysis of the energy profile of all seven complexes based on the **total interatomic** V_{inter}^{AB} **and intra-atomic energies** (E_{intra}^{A}) ranked by their REG values. The atomic labelling should be clear from the common core scheme X1-X2···N3-etc. Note that in Table 4 of the main text, which summarises this Table, X1 is referred to as X_{outer}, and X2 as X_{inner}.

HCN:F_2

SEG-1				SEG-2		
**TERM**	**REG**	**R**		**TERM**	**REG**	**R**
Intra_n3	6.36	0.98		Inter_f2_n3	6.47	0.94
Intra_f2	6.27	0.97		Intra_f1	3.48	0.97
Inter_f2_c4	1.43	0.98		Inter_n3_c4	0.54	0.98
Inter_f1_n3	1.02	0.99		Inter_f1_c4	0.45	0.86
Inter_f1_f2	0.99	0.99		Inter_n3_h5	0.25	1.00
Intra_c4	0.83	0.96		Inter_f1_h5	0.09	0.93
Inter_c4_h5	0.37	0.97		Inter_f1_n3	-0.06	-0.27
Inter_f2_h5	0.11	0.98		Intra_h5	-0.09	-0.99
Intra_h5	0.07	0.97		Inter_f2_h5	-0.10	-0.98
Inter_n3_h5	-0.13	-0.97		Inter_f1_f2	-0.15	-0.72
Inter_f1_h5	-0.15	-0.98		Inter_c4_h5	-0.51	-0.99
Inter_n3_c4	-0.70	-0.97		Intra_c4	-0.89	-0.99
Inter_f1_c4	-1.24	-0.98		Inter_f2_c4	-1.14	-0.98
Intra_f1	-3.39	-0.97		Intra_n3	-2.62	-0.90
Inter_f2_n3	-9.22	-0.97		Intra_f2	-5.93	-0.96

H_3N:F_2

SEG-1				SEG-2		
**TERM**	**REG**	**R**		**TERM**	**REG**	**R**
Intra_n3	6.65	0.99		Inter_f2_n3	7.46	0.94
Intra_f2	6.14	0.97		Intra_f1	4.17	0.97
Inter_f1_f2	3.48	1.00		Inter_f1_h6	0.34	0.91
Inter_f1_n3	1.83	0.99		Inter_f1_h4	0.34	0.91
Inter_f2_h6	0.54	0.99		Inter_f1_h5	0.34	0.91
Inter_f2_h5	0.54	0.99		Inter_n3_h6	0.01	0.12
Inter_f2_h4	0.54	0.99		Inter_n3_h5	0.01	0.12
Intra_h5	0.54	1.00		Inter_n3_h4	0.01	0.12

Intra_h6	0.54	1.00		Intra_h5	-0.17	-0.98
Intra_h4	0.54	1.00		Intra_h6	-0.17	-0.98
Inter_h4_h6	0.53	1.00		Intra_h4	-0.17	-0.98
Inter_h5_h6	0.53	1.00		Inter_h5_h6	-0.18	-0.97
Inter_h4_h5	0.53	1.00		Inter_h4_h5	-0.18	-0.97
Inter_n3_h4	-0.37	-0.97		Inter_h4_h6	-0.18	-0.97
Inter_n3_h6	-0.37	-0.97		Inter_f2_h4	-0.37	-0.97
Inter_n3_h5	-0.37	-0.97		Inter_f2_h6	-0.37	-0.97
Inter_f1_h4	-0.88	-0.99		Inter_f2_h5	-0.37	-0.97
Inter_f1_h5	-0.88	-0.99		Inter_f1_n3	-0.48	-0.82
Inter_f1_h6	-0.88	-0.99		Inter_f1_f2	-0.82	-0.86
Intra_f1	-5.04	-0.98		Intra_n3	-3.25	-0.93
Inter_f2_n3	-10.34	-0.98		Intra_f2	-6.55	-0.96

HCN:Cl_2

SEG-1				SEG-2		
**TERM**	**REG**	**R**		**TERM**	**REG**	**R**
Intra_cl2	6.50	0.97		Inter_cl2_n3	11.87	0.96
Intra_n3	6.35	0.98		Inter_n3_c4	4.34	0.98
Inter_cl2_c4	3.60	0.98		Intra_cl1	3.40	0.96
Intra_c4	1.75	0.97		Inter_cl1_c4	0.97	0.93
Inter_cl1_n3	1.66	0.99		Inter_n3_h5	0.86	0.98
Inter_c4_h5	0.96	0.97		Inter_cl1_h5	0.14	0.94
Inter_cl1_cl2	0.32	1.00		Inter_cl1_cl2	0.01	0.40
Inter_cl2_h5	0.30	0.98		Intra_h5	-0.26	-0.98
Intra_h5	0.21	0.98		Inter_cl2_h5	-0.33	-0.97
Inter_cl1_h5	-0.20	-0.98		Inter_cl1_n3	-0.78	-0.90
Inter_n3_h5	-0.58	-0.97		Inter_c4_h5	-1.28	-0.98
Inter_cl1_c4	-1.67	-0.98		Intra_c4	-2.47	-0.98
Inter_n3_c4	-2.58	-0.97		Inter_cl2_c4	-4.05	-0.97
Intra_cl1	-3.46	-0.98		Intra_n3	-5.72	-0.96
Inter_cl2_n3	-11.35	-0.97		Intra_cl2	-6.60	-0.95

H_3N:Cl_2

SEG-1				SEG-2		
**TERM**	**REG**	**R**		**TERM**	**REG**	**R**
Intra_n3	7.78	0.98		Inter_cl2_n3	10.54	0.94
Intra_cl2	6.30	0.96		Intra_cl1	3.58	0.94
Inter_cl1_n3	2.58	0.98		Inter_n3_h5	1.23	0.97

Inter_cl1_cl2	1.72	0.99		Inter_n3_h4	1.23	0.97
Inter_cl2_h6	1.51	0.98		Inter_n3_h6	1.23	0.97
Inter_cl2_h5	1.51	0.98		Inter_cl1_h6	0.46	0.89
Inter_cl2_h4	1.51	0.98		Inter_cl1_h5	0.46	0.89
Inter_h5_h6	0.97	0.98		Inter_cl1_h4	0.46	0.89
Inter_h4_h6	0.97	0.98		Inter_cl1_cl2	-0.51	-0.84
Inter_h4_h5	0.97	0.98		Intra_h6	-0.53	-0.94
Intra_h6	0.97	0.98		Intra_h5	-0.53	-0.94
Intra_h4	0.97	0.98		Intra_h4	-0.53	-0.94
Intra_h5	0.97	0.98		Inter_h5_h6	-0.56	-0.94
Inter_cl1_h4	-1.12	-0.98		Inter_h4_h5	-0.56	-0.94
Inter_cl1_h5	-1.12	-0.98		Inter_h4_h6	-0.56	-0.94
Inter_cl1_h6	-1.12	-0.98		Inter_cl1_n3	-1.04	-0.88
Inter_n3_h4	-1.59	-0.98		Inter_cl2_h4	-1.08	-0.96
Inter_n3_h5	-1.59	-0.98		Inter_cl2_h6	-1.08	-0.96
Inter_n3_h6	-1.59	-0.98		Inter_cl2_h5	-1.08	-0.96
Intra_cl1	-4.97	-0.97		Intra_n3	-4.91	-0.94
Inter_cl2_n3	-13.74	-0.97		Intra_cl2	-5.95	-0.94

N_2:ClF

SEG-1				SEG-2		
**TERM**	**REG**	**R**		**TERM**	**REG**	**R**
Intra_n3	5.56	0.98		Inter_cl2_n3	9.93	0.96
Intra_cl2	4.59	0.97		Inter_f1_cl2	1.21	0.98
Intra_n4	1.17	0.97		Inter_f1_n4	1.03	0.98
Inter_cl2_n4	1.06	0.96		Intra_f1	0.53	0.89
Inter_n3_n4	0.33	0.97		Inter_n3_n4	-0.36	-0.97
Inter_f1_n3	0.06	0.74		Inter_f1_n3	-0.84	-1.00
Inter_f1_cl2	-0.43	-0.89		Intra_n4	-1.83	-0.98
Inter_f1_n4	-0.58	-0.96		Inter_cl2_n4	-1.94	-0.98
Intra_f1	-1.16	-0.99		Intra_n3	-2.82	-0.91
Inter_cl2_n3	-8.71	-0.97		Intra_cl2	-4.86	-0.95

HCN:ClF

SEG-1				SEG-2		
**TERM**	**REG**	**R**		**TERM**	**REG**	**R**
Intra_n3	7.81	0.98		Inter_cl2_n3	13.03	0.97
Inter_cl2_c4	4.01	0.97		Inter_n3_c4	4.35	0.98

Intra_cl2	3.14	0.97		Inter_f1_c4	3.39	0.99
Intra_c4	2.42	0.97		Intra_f1	0.91	0.94
Inter_f1_n3	2.03	0.96		Inter_n3_h5	0.84	0.98
Inter_f1_cl2	1.39	1.00		Inter_f1_h5	0.40	0.99
Inter_c4_h5	1.28	0.97		Inter_f1_cl2	0.10	0.57
Inter_cl2_h5	0.39	0.97		Intra_h5	-0.27	-0.97
Intra_h5	0.30	0.98		Inter_cl2_h5	-0.54	-0.99
Inter_f1_h5	-0.31	-0.97		Inter_c4_h5	-1.30	-0.98
Inter_n3_h5	-0.75	-0.97		Intra_c4	-2.68	-0.97
Intra_f1	-1.72	-0.98		Intra_cl2	-3.19	-0.96
Inter_f1_c4	-2.22	-0.97		Inter_f1_n3	-3.93	-0.99
Inter_n3_c4	-3.25	-0.96		Intra_n3	-4.89	-0.95
Inter_cl2_n3	-12.71	-0.97		Inter_cl2_c4	-5.81	-0.99

H_3N:ClF

SEG-1				SEG-2		
**TERM**	**REG**	**R**		**TERM**	**REG**	**R**
Intra_n3	7.15	0.96		Inter_cl2_n3	9.80	0.97
Inter_f1_cl2	4.09	0.96		Intra_f1	1.27	0.93
Inter_f1_n3	1.47	0.96		Inter_n3_h4	1.27	0.97
Inter_cl2_h4	1.28	0.97		Inter_n3_h5	1.27	0.97
Inter_cl2_h5	1.28	0.97		Inter_n3_h6	1.27	0.97
Inter_cl2_h6	1.28	0.97		Inter_f1_h6	0.87	0.98
Intra_h6	0.92	0.96		Inter_f1_h4	0.87	0.98
Intra_h5	0.92	0.96		Inter_f1_h5	0.87	0.98
Intra_h4	0.92	0.96		Intra_h5	-0.64	-0.95
Inter_h5_h6	0.91	0.96		Intra_h4	-0.64	-0.95
Inter_h4_h6	0.91	0.96		Intra_h6	-0.64	-0.95
Inter_h4_h5	0.91	0.96		Inter_h4_h5	-0.66	-0.95
Intra_cl2	0.39	0.99		Inter_h5_h6	-0.66	-0.95
Inter_f1_h4	-0.98	-0.96		Inter_h4_h6	-0.66	-0.95
Inter_f1_h6	-0.98	-0.96		Inter_cl2_h4	-1.22	-0.99
Inter_f1_h5	-0.98	-0.96		Inter_cl2_h6	-1.22	-0.99
Inter_n3_h6	-1.24	-0.95		Inter_cl2_h5	-1.22	-0.99
Inter_n3_h5	-1.24	-0.95		Intra_cl2	-1.30	-1.00
Inter_n3_h4	-1.24	-0.95		Inter_f1_cl2	-1.76	-0.88
Intra_f1	-2.17	-0.96		Inter_f1_n3	-2.04	-0.99
Inter_cl2_n3	-12.11	-0.97		Intra_n3	-4.41	-0.94

Table S4

Level 3 REG analysis of the energy profile of all seven complexes based **on the total intra-atomic energy E_{intra}^{A}, and the interatomic atomic energies V_{cl} (electrostatic), V_x (exchange) and V_c correlation (overall 4 possible energy types)**, ranked by their REG values. The atomic labelling should be clear from the common core scheme X1-X2···N3-etc. Note that in Table 5 of the main text, which summarises this Table, X1 is referred to as X_{outer}, and X2 as X_{inner}.

HCN:F_2

SEG-1				SEG-2		
**TERM**	**REG**	**R**		**TERM**	**REG**	**R**
Intra_n3	6.36	0.98		Vx_f2_n3	4.81	0.93
Intra_f2	6.27	0.97		Intra_f1	3.48	0.97
Vx_f1_f2	2.52	0.98		Vcl_f2_n3	1.66	0.97
Vcl_f2_c4	1.57	0.98		Vcl_n3_c4	0.94	0.99
Vcl_f1_n3	1.44	0.98		Vcl_f1_f2	0.94	0.95
Intra_c4	0.83	0.96		Vc_f2_n3	0.89	0.97
Vx_n3_c4	0.46	0.97		Vcl_f1_c4	0.44	0.86
Vcl_c4_h5	0.30	0.97		Vcl_n3_h5	0.30	1.00
Vc_f1_n3	0.18	0.96		Vx_f1_n3	0.26	0.93
Vcl_f2_h5	0.12	0.98		Vc_f1_c4	0.16	0.98
Vc_f2_c4	0.10	0.95		Vx_f2_c4	0.11	0.94
Vx_c4_h5	0.08	0.98		Vcl_f1_h5	0.09	0.93
Vc_n3_c4	0.07	0.97		Vc_f1_h5	0.03	0.99
Intra_h5	0.07	0.97		Vx_f2_h5	0.01	0.96
Vx_n3_h5	0.03	0.96		Vx_f1_c4	0.01	0.88
Vc_n3_h5	0.03	0.97		Vx_f1_h5	0.00	0.94
Vc_c4_h5	0.01	0.96		Vc_c4_h5	-0.02	-0.99
Vc_f2_h5	0.00	-0.03		Vc_n3_h5	-0.02	-0.90
Vx_f1_h5	0.00	-0.98		Vc_f1_f2	-0.02	-0.98
Vx_f2_h5	-0.01	-0.96		Vc_f2_h5	-0.02	-1.00
Vx_f1_c4	-0.02	-0.98		Vx_n3_h5	-0.04	-0.98
Vc_f1_h5	-0.02	-0.95		Vc_n3_c4	-0.04	-0.87
Vc_f1_f2	-0.07	-1.00		Vx_c4_h5	-0.08	-0.98
Vc_f1_c4	-0.11	-0.96		Intra_h5	-0.09	-0.99
Vx_f2_c4	-0.15	-0.97		Vcl_f2_h5	-0.11	-0.98
Vcl_f1_h5	-0.15	-0.98		Vc_f2_c4	-0.18	-0.99
Vcl_n3_h5	-0.16	-0.97		Vc_f1_n3	-0.27	-0.98
Vx_f1_n3	-0.42	-0.97		Vcl_f1_n3	-0.31	-0.72
Vc_f2_n3	-0.75	-0.96		Vx_n3_c4	-0.40	-0.98

Vcl_n3_c4	-1.16	-0.97		Vcl_c4_h5	-0.43	-0.99
Vcl_f1_c4	-1.22	-0.98		Intra_c4	-0.89	-0.99
Vcl_f1_f2	-1.54	-0.98		Vx_f1_f2	-1.09	-0.92
Vcl_f2_n3	-2.02	-0.98		Vcl_f2_c4	-1.26	-0.97
Intra_f1	-3.39	-0.97		Intra_n3	-2.62	-0.90
Vx_f2_n3	-7.20	-0.97		Intra_f2	-5.93	-0.96

$H_3N:F_2$

SEG-1				SEG-2		
**TERM**	**REG**	**R**		**TERM**	**REG**	**R**
Vx_f1_f2	6.80	0.99		Vx_f2_n3	5.65	0.93
Intra_n3	6.65	0.99		Intra_f1	4.17	0.97
Intra_f2	6.14	0.97		Vcl_f2_n3	1.81	0.96
Vcl_f1_n3	2.61	0.99		Vcl_f1_f2	1.60	0.93
Vcl_f2_h6	0.62	0.99		Vc_f2_n3	0.60	0.97
Vcl_f2_h5	0.62	0.99		Vx_f1_n3	0.36	0.92
Vcl_f2_h4	0.62	0.99		Vcl_f1_h6	0.33	0.91
Vx_n3_h6	0.61	1.00		Vcl_f1_h4	0.33	0.91
Vx_n3_h4	0.61	1.00		Vcl_f1_h5	0.33	0.91
Vx_n3_h5	0.61	1.00		Vcl_n3_h4	0.26	0.98
Intra_h5	0.54	1.00		Vcl_n3_h6	0.26	0.99
Intra_h6	0.54	1.00		Vcl_n3_h5	0.26	0.98
Intra_h4	0.54	1.00		Vc_f1_f2	0.06	0.78
Vcl_h4_h6	0.51	1.00		Vx_f2_h4	0.06	0.94
Vcl_h5_h6	0.51	1.00		Vx_f2_h5	0.06	0.94
Vcl_h4_h5	0.51	1.00		Vx_f2_h6	0.06	0.94
Vc_f1_n3	0.21	0.99		Vc_f1_h5	0.05	0.97
Vc_n3_h4	0.06	0.96		Vc_f1_h6	0.05	0.97
Vc_n3_h5	0.06	0.96		Vc_f1_h4	0.05	0.97
Vc_n3_h6	0.06	0.96		Vx_f1_h5	0.01	0.89
Vx_h4_h5	0.02	1.00		Vx_f1_h4	0.01	0.89
Vx_h4_h6	0.02	1.00		Vx_f1_h6	0.01	0.89
Vx_h5_h6	0.02	1.00		Vc_h5_h6	0.00	-0.61
Vc_h4_h6	0.01	1.00		Vc_h4_h6	0.00	-0.61
Vc_h5_h6	0.01	1.00		Vc_h4_h5	0.00	-0.61
Vc_h4_h5	0.01	1.00		Vc_f2_h4	0.00	-0.29
Vx_f1_h6	-0.02	-0.99		Vc_f2_h5	0.00	-0.29
Vx_f1_h4	-0.02	-0.99		Vc_f2_h6	0.00	-0.29
Vx_f1_h5	-0.02	-0.99		Vx_h5_h6	-0.01	-0.94
Vc_f2_h5	-0.03	-0.99		Vx_h4_h5	-0.01	-0.94
Vc_f2_h6	-0.03	-0.99		Vx_h4_h6	-0.01	-0.94

Vc_f2_h4	-0.03	-0.99		Vc_n3_h4	-0.06	-0.95
Vc_f1_h5	-0.05	-0.97		Vc_n3_h5	-0.06	-0.95
Vc_f1_h6	-0.05	-0.97		Vc_n3_h6	-0.06	-0.95
Vc_f1_h4	-0.05	-0.97		Vcl_h5_h6	-0.17	-0.98
Vx_f2_h6	-0.07	-0.98		Vcl_h4_h5	-0.17	-0.98
Vx_f2_h5	-0.07	-0.98		Vcl_h4_h6	-0.17	-0.98
Vx_f2_h4	-0.07	-0.98		Intra_h5	-0.17	-0.98
Vc_f2_n3	-0.43	-0.95		Intra_h6	-0.17	-0.98
Vc_f1_f2	-0.43	-1.00		Intra_h4	-0.17	-0.98
Vx_f1_n3	-0.78	-0.99		Vc_f1_n3	-0.18	-0.97
Vcl_f1_h4	-0.86	-0.99		Vx_n3_h5	-0.25	-0.96
Vcl_f1_h5	-0.86	-0.99		Vx_n3_h6	-0.25	-0.96
Vcl_f1_h6	-0.86	-0.99		Vx_n3_h4	-0.25	-0.96
Vcl_n3_h4	-0.98	-1.00		Vcl_f2_h4	-0.42	-0.97
Vcl_n3_h6	-0.98	-1.00		Vcl_f2_h6	-0.42	-0.97
Vcl_n3_h5	-0.98	-1.00		Vcl_f2_h5	-0.42	-0.97
Vcl_f2_n3	-2.28	-0.98		Vcl_f1_n3	-0.84	-0.87
Vcl_f1_f2	-3.32	-0.99		Vx_f1_f2	-2.42	-0.91
Intra_f1	-5.04	-0.98		Intra_n3	-3.25	-0.93
Vx_f2_n3	-8.06	-0.98		Intra_f2	-6.55	-0.96

HCN:Cl_2

SEG-1				SEG-2		
**TERM**	**REG**	**R**		**TERM**	**REG**	**R**
Intra_cl2	6.50	0.97		Vx_cl2_n3	6.07	0.95
Intra_n3	6.35	0.98		Vcl_cl2_n3	5.80	0.97
Vcl_cl2_c4	3.76	0.98		Vcl_n3_c4	5.43	0.98
Vcl_cl1_n3	1.94	0.98		Intra_cl1	3.40	0.96
Intra_c4	1.75	0.97		Vcl_cl1_c4	0.96	0.93
Vx_cl1_cl2	1.68	0.99		Vcl_n3_h5	0.93	0.98
Vx_n3_c4	0.97	0.98		Vc_cl2_n3	0.88	0.97
Vcl_c4_h5	0.78	0.97		Vcl_cl1_cl2	0.83	0.95
Vcl_cl2_h5	0.31	0.98		Vx_cl1_n3	0.25	0.96
Intra_h5	0.21	0.98		Vx_cl2_c4	0.14	0.95
Vx_c4_h5	0.18	0.98		Vcl_cl1_h5	0.14	0.94
Vc_cl1_n3	0.11	0.97		Vc_cl1_c4	0.11	0.98
Vx_n3_h5	0.06	0.97		Vc_n3_c4	0.03	0.99
Vc_cl2_c4	0.05	0.94		Vc_cl1_h5	0.02	0.98
Vc_n3_h5	0.02	0.98		Vx_cl1_c4	0.01	0.91
Vc_c4_h5	0.02	0.97		Vx_cl2_h5	0.01	0.96
Vc_n3_c4	0.00	0.69		Vc_cl1_cl2	0.01	0.49
Vx_cl1_h5	0.00	-0.98		Vx_cl1_h5	0.00	0.91

Vc_cl2_h5	-0.01	-1.00		Vc_cl2_h5	-0.01	-0.99
Vx_cl2_h5	-0.01	-0.97		Vc_n3_h5	-0.01	-0.91
Vc_cl1_h5	-0.01	-0.96		Vc_c4_h5	-0.03	-0.98
Vx_cl1_c4	-0.02	-0.99		Vx_n3_h5	-0.08	-0.97
Vc_cl1_c4	-0.06	-0.96		Vc_cl2_c4	-0.14	-0.99
Vc_cl1_cl2	-0.09	-1.00		Vc_cl1_n3	-0.17	-0.98
Vx_cl2_c4	-0.16	-0.98		Vx_c4_h5	-0.21	-0.98
Vcl_cl1_h5	-0.20	-0.98		Intra_h5	-0.26	-0.98
Vx_cl1_n3	-0.28	-0.98		Vcl_cl2_h5	-0.34	-0.97
Vc_cl2_n3	-0.61	-0.96		Vx_cl1_cl2	-0.82	-0.93
Vcl_n3_h5	-0.64	-0.97		Vcl_cl1_n3	-1.03	-0.91
Vcl_cl1_cl2	-1.36	-0.99		Vcl_c4_h5	-1.07	-0.98
Vcl_cl1_c4	-1.65	-0.98		Vx_n3_c4	-1.09	-0.97
Intra_cl1	-3.46	-0.98		Intra_c4	-2.47	-0.98
Vcl_n3_c4	-3.56	-0.97		Vcl_cl2_c4	-4.19	-0.97
Vcl_cl2_n3	-4.91	-0.97		Intra_n3	-5.72	-0.96
Vx_cl2_n3	-6.43	-0.97		Intra_cl2	-6.60	-0.95

$H_3N:Cl_2$

SEG-1				SEG-2		
**TERM**	**REG**	**R**		**TERM**	**REG**	**R**
Intra_n3	7.78	0.98		Vx_cl2_n3	5.80	0.93
Intra_cl2	6.30	0.96		Vcl_cl2_n3	4.74	0.96
Vx_cl1_cl2	4.74	0.99		Intra_cl1	3.58	0.94
Vcl_cl1_n3	3.08	0.98		Vcl_n3_h5	1.84	0.96
Vcl_cl2_h6	1.60	0.98		Vcl_n3_h4	1.84	0.96
Vcl_cl2_h5	1.60	0.98		Vcl_n3_h6	1.84	0.96
Vcl_cl2_h4	1.60	0.98		Vcl_cl1_cl2	1.27	0.90
Vx_n3_h4	1.05	0.98		Vcl_cl1_h6	0.46	0.89
Vx_n3_h6	1.05	0.98		Vcl_cl1_h5	0.46	0.89
Vx_n3_h5	1.05	0.98		Vcl_cl1_h4	0.46	0.89
Intra_h6	0.97	0.98		Vc_cl2_n3	0.45	0.97
Intra_h4	0.97	0.98		Vx_cl1_n3	0.30	0.92
Intra_h5	0.97	0.98		Vc_cl1_cl2	0.07	0.79
Vcl_h5_h6	0.93	0.98		Vx_cl2_h5	0.06	0.92
Vcl_h4_h6	0.93	0.98		Vx_cl2_h4	0.06	0.92
Vcl_h4_h5	0.93	0.98		Vx_cl2_h6	0.06	0.92
Vc_cl1_n3	0.13	0.97		Vc_cl1_h5	0.03	0.96
Vx_h4_h6	0.04	0.98		Vc_cl1_h6	0.03	0.96
Vx_h4_h5	0.04	0.98		Vc_cl1_h4	0.03	0.96
Vx_h5_h6	0.04	0.98		Vc_cl2_h4	0.02	0.83

Vc_n3_h4	0.03	0.92		Vc_cl2_h5	0.02	0.83
Vc_n3_h5	0.03	0.92		Vc_cl2_h6	0.02	0.83
Vc_n3_h6	0.03	0.92		Vx_cl1_h5	0.01	0.89
Vc_h5_h6	0.01	0.96		Vx_cl1_h4	0.01	0.89
Vc_h4_h5	0.01	0.96		Vx_cl1_h6	0.01	0.89
Vc_h4_h6	0.01	0.96		Vc_h5_h6	-0.01	-0.93
Vx_cl1_h5	-0.01	-0.98		Vc_h4_h5	-0.01	-0.93
Vx_cl1_h4	-0.01	-0.98		Vc_h4_h6	-0.01	-0.93
Vx_cl1_h6	-0.01	-0.98		Vx_h4_h6	-0.02	-0.94
Vc_cl1_h4	-0.02	-0.95		Vx_h5_h6	-0.02	-0.94
Vc_cl1_h5	-0.02	-0.95		Vx_h4_h5	-0.02	-0.94
Vc_cl1_h6	-0.02	-0.95		Vc_n3_h5	-0.04	-0.92
Vc_cl2_h5	-0.05	-0.97		Vc_n3_h6	-0.04	-0.92
Vc_cl2_h6	-0.05	-0.97		Vc_n3_h4	-0.04	-0.92
Vc_cl2_h4	-0.05	-0.97		Vc_cl1_n3	-0.10	-0.95
Vx_cl2_h4	-0.09	-0.98		Intra_h6	-0.53	-0.94
Vx_cl2_h6	-0.09	-0.98		Intra_h5	-0.53	-0.94
Vx_cl2_h5	-0.09	-0.98		Intra_h4	-0.53	-0.94
Vc_cl2_n3	-0.25	-0.92		Vcl_h5_h6	-0.54	-0.95
Vc_cl1_cl2	-0.33	-0.99		Vcl_h4_h5	-0.54	-0.95
Vx_cl1_n3	-0.51	-0.97		Vcl_h4_h6	-0.54	-0.95
Vcl_cl1_h4	-1.10	-0.98		Vx_n3_h5	-0.61	-0.94
Vcl_cl1_h5	-1.10	-0.98		Vx_n3_h4	-0.61	-0.94
Vcl_cl1_h6	-1.10	-0.98		Vx_n3_h6	-0.61	-0.94
Vcl_n3_h4	-2.64	-0.98		Vcl_cl2_h4	-1.14	-0.95
Vcl_n3_h5	-2.64	-0.98		Vcl_cl2_h6	-1.14	-0.95
Vcl_n3_h6	-2.64	-0.98		Vcl_cl2_h5	-1.14	-0.95
Vcl_cl1_cl2	-3.02	-0.98		Vcl_cl1_n3	-1.34	-0.89
Intra_cl1	-4.97	-0.97		Vx_cl1_cl2	-1.78	-0.89
Vcl_cl2_n3	-5.52	-0.97		Intra_n3	-4.91	-0.94
Vx_cl2_n3	-8.22	-0.97		Intra_cl2	-5.95	-0.94

N_2:ClF

SEG-1				SEG-2		
**TERM**	**REG**	**R**		**TERM**	**REG**	**R**
Intra_n3	5.56	0.98		Vx_cl2_n3	6.98	0.95
Intra_cl2	4.59	0.97		Vcl_cl2_n3	2.95	0.99
Vx_f1_cl2	2.01	0.98		Vcl_f1_cl2	2.59	0.96
Vcl_cl2_n4	1.36	0.97		Vc_cl2_n3	1.05	0.97
Intra_n4	1.17	0.97		Vcl_f1_n4	1.01	0.98
Vx_n3_n4	0.50	0.99		Intra_f1	0.53	0.89

Vcl_f1_n3	0.31	0.94		Vx_cl2_n4	0.28	0.95
Vc_n3_n4	0.24	0.96		Vx_f1_n3	0.25	0.96
Vc_f1_n3	0.12	0.96		Vc_f1_n4	0.17	0.98
Vc_cl2_n4	0.04	0.90		Vc_f1_cl2	0.13	0.95
Vx_f1_n4	-0.03	-0.98		Vx_f1_n4	0.01	0.91
Vc_f1_n4	-0.09	-0.95		Vcl_n3_n4	-0.09	-0.98
Vcl_n3_n4	-0.17	-1.00		Vc_cl2_n4	-0.20	-0.99
Vc_f1_cl2	-0.19	-0.98		Vc_f1_n3	-0.22	-0.98
Vx_f1_n3	-0.25	-0.97		Vx_n3_n4	-0.27	-0.92
Vx_cl2_n4	-0.30	-0.97		Vc_n3_n4	-0.29	-0.96
Vcl_f1_n4	-0.55	-0.96		Vcl_f1_n3	-1.09	-0.99
Vc_cl2_n3	-0.69	-0.96		Vx_f1_cl2	-1.38	-0.94
Vcl_cl2_n3	-1.10	-0.94		Intra_n4	-1.83	-0.98
Intra_f1	-1.16	-0.99		Vcl_cl2_n4	-2.21	-0.98
Vcl_f1_cl2	-2.44	-0.97		Intra_n3	-2.82	-0.91
Vx_cl2_n3	-7.61	-0.97		Intra_cl2	-4.86	-0.95

HCN:ClF

SEG-1				SEG-2		
**TERM**	**REG**	**R**		**TERM**	**REG**	**R**
Intra_n3	7.81	0.98		Vcl_cl2_n3	7.93	0.99
Vcl_cl2_c4	4.22	0.97		Vcl_n3_c4	5.50	0.98
Intra_cl2	3.14	0.97		Vx_cl2_n3	5.10	0.94
Vx_f1_cl2	3.12	0.98		Vcl_f1_c4	3.38	0.99
Intra_c4	2.42	0.97		Vcl_f1_cl2	1.78	0.97
Vcl_f1_n3	2.30	0.96		Intra_f1	0.91	0.94
Vx_n3_c4	1.40	0.98		Vcl_n3_h5	0.91	0.98
Vcl_c4_h5	1.04	0.97		Vc_cl2_n3	0.57	0.97
Vcl_cl2_h5	0.40	0.97		Vcl_f1_h5	0.40	0.99
Intra_h5	0.30	0.98		Vx_f1_n3	0.17	0.94
Vx_c4_h5	0.23	0.98		Vc_f1_cl2	0.14	0.94
Vc_f1_n3	0.09	0.97		Vx_cl2_c4	0.12	0.94
Vx_n3_h5	0.07	0.97		Vc_n3_c4	0.07	0.99
Vc_n3_h5	0.02	0.97		Vc_f1_c4	0.06	0.98
Vc_c4_h5	0.02	0.96		Vc_f1_h5	0.01	0.98
Vc_cl2_c4	0.01	0.94		Vx_f1_c4	0.01	0.90
Vx_f1_h5	0.00	-0.98		Vx_cl2_h5	0.01	0.95
Vc_f1_h5	-0.01	-0.95		Vc_cl2_h5	0.00	0.41
Vx_cl2_h5	-0.01	-0.98		Vx_f1_h5	0.00	0.91
Vc_cl2_h5	-0.02	-0.98		Vc_n3_h5	-0.01	-0.86
Vx_f1_c4	-0.02	-0.99		Vc_c4_h5	-0.03	-0.98

Vc_f1_c4	-0.04	-0.95		Vc_cl2_c4	-0.05	-1.00
Vc_n3_c4	-0.08	-0.99		Vx_n3_h5	-0.07	-0.97
Vx_cl2_c4	-0.21	-0.98		Vc_f1_n3	-0.10	-0.98
Vc_f1_cl2	-0.25	-0.98		Vx_c4_h5	-0.21	-0.97
Vx_f1_n3	-0.27	-0.98		Intra_h5	-0.27	-0.97
Vcl_f1_h5	-0.31	-0.97		Vcl_cl2_h5	-0.55	-0.99
Vc_cl2_n3	-0.40	-0.94		Vcl_c4_h5	-1.08	-0.98
Vcl_n3_h5	-0.82	-0.97		Vx_n3_c4	-1.15	-0.97
Intra_f1	-1.72	-0.98		Vx_f1_cl2	-1.68	-0.94
Vcl_f1_cl2	-1.73	-0.97		Intra_c4	-2.68	-0.97
Vcl_f1_c4	-2.20	-0.97		Intra_cl2	-3.19	-0.96
Vcl_n3_c4	-4.65	-0.96		Vcl_f1_n3	-4.10	-0.99
Vcl_cl2_n3	-5.07	-0.97		Intra_n3	-4.89	-0.95
Vx_cl2_n3	-7.64	-0.98		Vcl_cl2_c4	-5.93	-0.99

H_3N:ClF

SEG-1				SEG-2		
**TERM**	**REG**	**R**		**TERM**	**REG**	**R**
Intra_n3	7.15	0.96		Vx_cl2_n3	5.33	0.94
Vx_f1_cl2	4.43	0.96		Vcl_cl2_n3	4.47	0.99
Vcl_f1_n3	1.70	0.96		Vcl_n3_h6	1.96	0.97
Vcl_cl2_h4	1.37	0.97		Vcl_n3_h4	1.96	0.97
Vcl_cl2_h5	1.37	0.97		Vcl_n3_h5	1.96	0.97
Vcl_cl2_h6	1.37	0.97		Intra_f1	1.27	0.93
Vx_n3_h5	0.98	0.96		Vcl_f1_h6	0.87	0.98
Vx_n3_h6	0.98	0.96		Vcl_f1_h4	0.87	0.98
Vx_n3_h4	0.98	0.96		Vcl_f1_h5	0.87	0.98
Intra_h6	0.92	0.96		Vcl_f1_cl2	0.73	1.00
Intra_h5	0.92	0.96		Vc_cl2_n3	0.24	0.99
Intra_h4	0.92	0.96		Vx_f1_n3	0.23	0.93
Vcl_h5_h6	0.88	0.96		Vc_f1_cl2	0.19	0.92
Vcl_h4_h6	0.88	0.96		Vx_cl2_h5	0.06	0.93
Vcl_h4_h5	0.88	0.96		Vx_cl2_h4	0.06	0.93
Intra_cl2	0.39	0.99		Vx_cl2_h6	0.06	0.93
Vc_cl2_n3	0.07	0.95		Vc_cl2_h4	0.03	0.92
Vc_f1_n3	0.07	0.91		Vc_cl2_h5	0.03	0.92
Vx_h5_h6	0.03	0.95		Vc_cl2_h6	0.03	0.92
Vx_h4_h5	0.03	0.95		Vc_f1_h5	0.02	0.98
Vx_h4_h6	0.03	0.95		Vc_f1_h6	0.02	0.98
Vc_h5_h6	0.00	0.85		Vc_f1_h4	0.02	0.98
Vc_h4_h5	0.00	0.85		Vx_f1_h5	0.01	0.91

Vc_h4_h6	0.00	0.85		Vx_f1_h4	0.01	0.91
Vc_n3_h6	0.00	0.61		Vx_f1_h6	0.01	0.91
Vc_n3_h5	0.00	0.61		Vc_h5_h6	-0.01	-0.96
Vc_n3_h4	0.00	0.58		Vc_h4_h5	-0.01	-0.96
Vx_f1_h5	-0.01	-0.94		Vc_h4_h6	-0.01	-0.96
Vx_f1_h6	-0.01	-0.94		Vc_n3_h6	-0.02	-0.93
Vx_f1_h4	-0.01	-0.94		Vc_n3_h5	-0.02	-0.93
Vc_f1_h5	-0.01	-0.88		Vc_n3_h4	-0.02	-0.93
Vc_f1_h6	-0.01	-0.88		Vx_h5_h6	-0.03	-0.96
Vc_f1_h4	-0.01	-0.88		Vx_h4_h6	-0.03	-0.96
Vc_cl2_h5	-0.04	-0.94		Vx_h4_h5	-0.03	-0.96
Vc_cl2_h6	-0.04	-0.94		Vc_f1_n3	-0.07	-0.95
Vc_cl2_h4	-0.04	-0.94		Vcl_h4_h5	-0.64	-0.95
Vx_cl2_h6	-0.09	-0.96		Vcl_h5_h6	-0.64	-0.95
Vx_cl2_h4	-0.09	-0.96		Vcl_h4_h6	-0.64	-0.95
Vx_cl2_h5	-0.09	-0.96		Intra_h5	-0.64	-0.95
Vx_f1_n3	-0.23	-0.91		Intra_h4	-0.64	-0.95
Vc_f1_cl2	-0.33	-0.96		Intra_h6	-0.64	-0.95
Vcl_f1_cl2	-0.34	-0.95		Vx_n3_h5	-0.69	-0.95
Vcl_f1_h4	-0.97	-0.96		Vx_n3_h4	-0.69	-0.95
Vcl_f1_h6	-0.97	-0.96		Vx_n3_h6	-0.69	-0.95
Vcl_f1_h5	-0.97	-0.96		Vcl_cl2_h4	-1.28	-0.99
Intra_f1	-2.17	-0.96		Vcl_cl2_h6	-1.28	-0.99
Vcl_n3_h6	-2.23	-0.96		Vcl_cl2_h5	-1.28	-0.99
Vcl_n3_h5	-2.23	-0.96		Intra_cl2	-1.30	-1.00
Vcl_n3_h4	-2.23	-0.96		Vcl_f1_n3	-2.27	-0.99
Vcl_cl2_n3	-3.61	-0.97		Vx_f1_cl2	-2.49	-0.92
Vx_cl2_n3	-8.50	-0.97		Intra_n3	-4.41	-0.94

Figure S3. Relation between the total energy of H_3N:ClF (relative to the energy minimum, in Hartree), and the electrostatic (red) and exchange (blue) energy contributions (relative to the energy minimum, in Hartree) for the (top) **full** complex formation energy segment (SEG2) and the (bottom) **long-range-only** part of the complex formation segment.

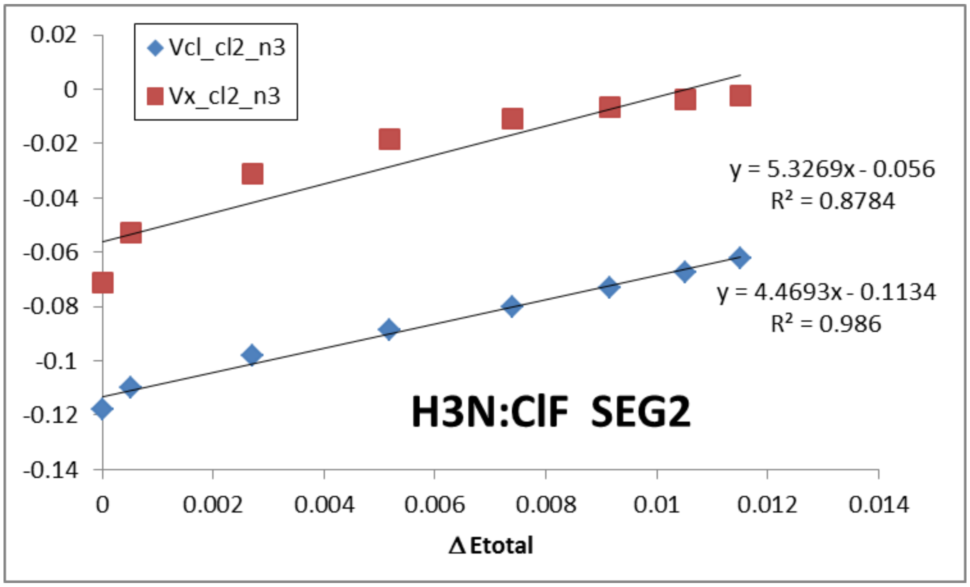

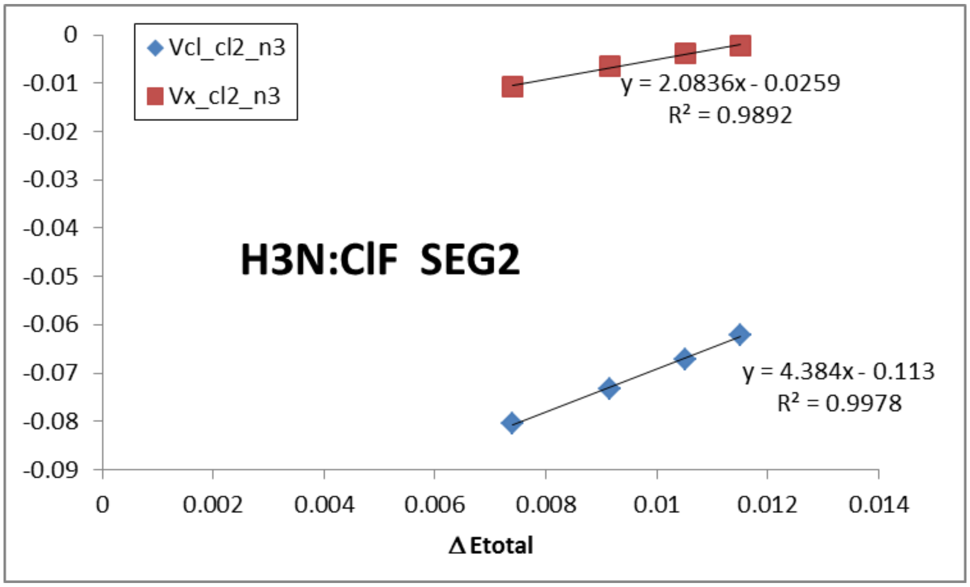

Figure S4. Charge transfer profiles for all 7 complexes. The overall net charge of the halogen molecule is plotted (in *e*) *versus* the N…X distance (in Å). The bold vertical line marks the equilibrium distance.

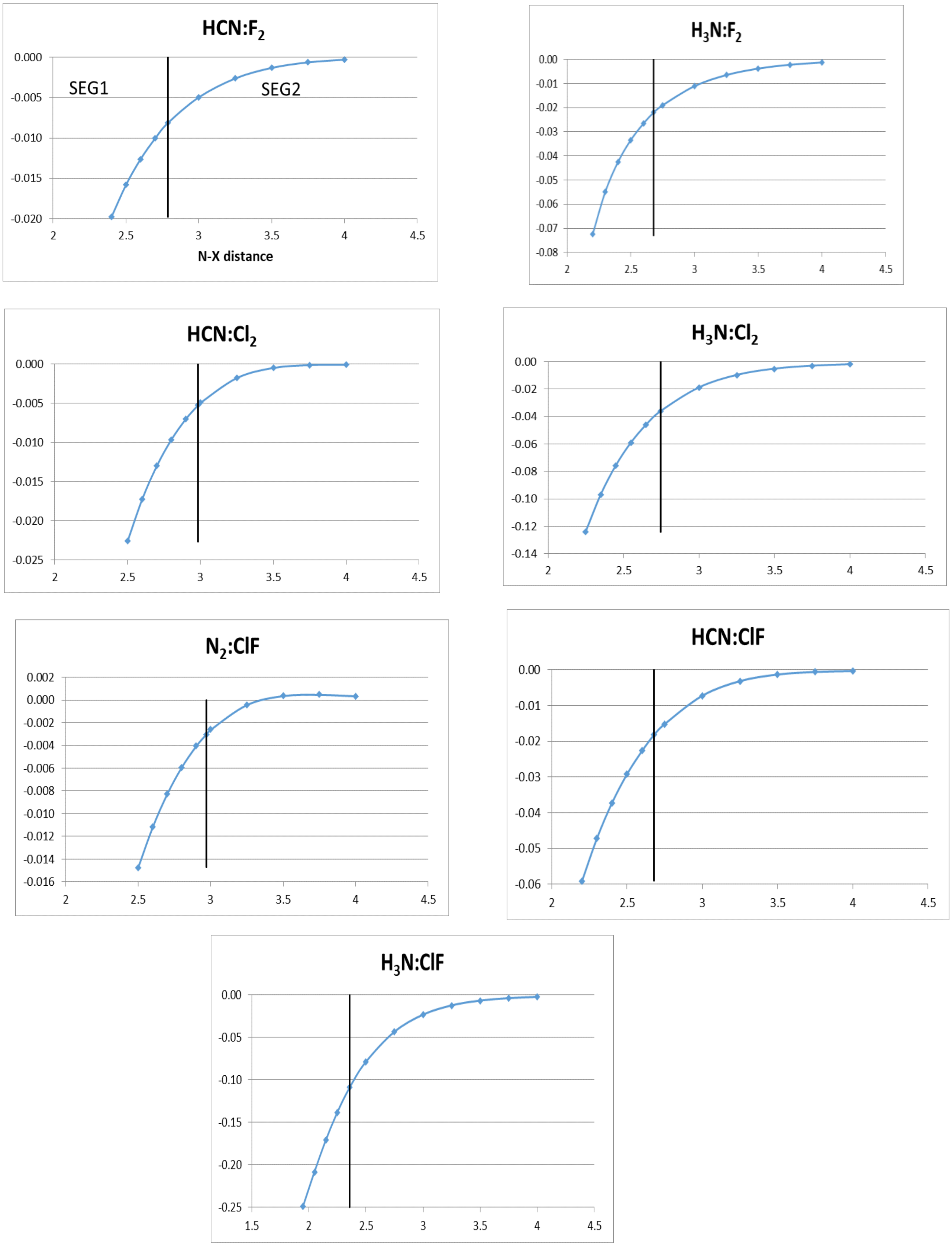

Supplement: Supplementary file 1 [file molecules-25-02674-s001.pdf]
